# Accuracy of pre-hospital triage tools for major trauma: a systematic review with meta-analysis and net clinical benefit

**DOI:** 10.1186/s13017-021-00372-1

**Published:** 2021-06-10

**Authors:** Silvia Gianola, Greta Castellini, Annalisa Biffi, Gloria Porcu, Andrea Fabbri, Maria Pia Ruggieri, Nino Stocchetti, Antonello Napoletano, Daniela Coclite, Daniela D’Angelo, Alice Josephine Fauci, Laura Iacorossi, Roberto Latina, Katia Salomone, Shailvi Gupta, Primiano Iannone, Osvaldo Chiara, the Italian National Institute of Health guideline working group, Carlo Coniglio, Elvio De Blasio, Gaddo Flego, Massimo Geraci, Giulio Maccauro, Antonio Rampoldi, Federico Santolini, Claudio Tacconi, Gregorio Tugnoli

**Affiliations:** 1 Unit of Clinical Epidemiology, IRCCS Istituto Ortopedico Galeazzi, Milan, Italy; 2 National Centre for Healthcare Research and Pharmacoepidemiology, Department of Statistics and Quantitative Methods, University of Milano-Bicocca, Milan, Italy; 3 Unit of Biostatistics, Epidemiology and Public Health, Department of Statistics and Quantitative Methods, University of Milano-Bicocca, Milan, Italy; 4 Emergency Department, AUSL della Romagna, Forlì, Italy; 5 Emergency Department, AO San Giovanni Addolorata, Rome, Italy; 6 Department of Pathophysiology and Transplantation, University of Milan, Milan, Italy; 7 Fondazione Istituto di Ricovero e Cura a Carattere Scientifico (IRCCS) Cà Granda-Ospedale Maggiore Policlinico, Milan, Italy; 8 Centro Eccellenza Clinica Qualità e Sicurezza delle Cure, Istituto Superiore di Sanità, Rome, Italy; 9 Adams Cowley Shock Trauma Center, University of Maryland, Baltimora, MD USA; 10 General Surgery and Trauma Team, ASST Grande Ospedale Metropolitano Niguarda, University of Milan, Piazza Ospedale Maggiore, Milan, Milano Italy

**Keywords:** Systematic review, Major trauma, Triage, Pre-hospital, Accuracy

## Abstract

**Background:**

We conducted a systematic review to evaluate and compare the accuracy of pre-hospital triage tools for major trauma in the context of the development of the Italian National Institute of Health guidelines on major trauma integrated management.

**Methods:**

PubMed, Embase, and CENTRAL were searched up to November 2019 for studies investigating pre-hospital triage tools. The ROC (receiver operating characteristics) curve and net clinical benefit for all selected triage tools were performed. Quality assessment was performed using the Quality Assessment of Diagnostic Accuracy Studies–2. Certainty of the evidence was judged with the Grading of Recommendations Assessment, Development and Evaluation (GRADE) approach.

**Results:**

We found 15 observational studies of 13 triage tools for adults and 11 for children. In adults, according to the ROC curve and the net clinical benefit, the most reliable tool was the Northern French Alps Trauma System (TRENAU), adopting injury severity score (ISS) > 15 as reference (sensitivity (Sn), 0.92; specificity (Sp), 0.41; 1 study; sample size, 2572; high certainty of the evidence). When mortality as reference was considered, the pre-hospital triage tool with the best net clinical benefit trajectory was the New Trauma Score (NTS) < 18 (Sn, 0.82; Sp, 0.86; 1 study; sample size, 1001; moderate certainty of the evidence). In children, high variability among all triage tools for sensitivity and specificity was found.

**Conclusion:**

Sensitivity and specificity varied across all available pre-hospital trauma triage tools. TRENAU and NTS are the best accurate triage tools for adults, whereas in the pediatric area a large variability prevents any firm conclusion.

**Supplementary Information:**

The online version contains supplementary material available at 10.1186/s13017-021-00372-1.

## Introduction

Severely injured trauma patients represent a global concern, responsible for over 5 million deaths each year and leaving even more patients with lifelong injury-related disabilities [1]. In order to improve a patient’s chance of survival, high level facilities dedicated to trauma care should be utilized [2, 3]. The American College of Surgeons Committee on Trauma (ACS-COT) recommends that severely injured trauma patients be treated at levels I and II trauma care facilities [4]. Pre-hospital estimation of one’s injury severity is essential for pre-hospital therapy, as it determines the destination hospital and the associated level of trauma care. In stressful situations and under great pressure, it can be difficult to make a correct estimate of injury severity [5]. In this context, pre-hospital trauma triage is a critical step in transporting the right patient to the right hospital in a timely fashion; this makes pre-hospital care imperative as an integrated part of the whole health-care pathway [6]. Pre-hospital trauma triage tools are generally based on a scoring system which leads to the transport of the patient toward the best treatment solution [1]. Patients with major trauma should be transported to a high-level facility for trauma care. Incorrect triage results in both under triage and over triage. Indeed, a low sensitivity triage tool indicates a significant number of false negative cases, which means the possible failure in diagnosis and treatment of a severe injury. Under triage has been associated with an increased mortality rate [5]. Conversely, a low specificity is associated with a high rate of false positive cases, i.e., over triage. Patients with minor injuries are admitted to high level of care facilities with unnecessary use of hospital resources and increased adverse events of concurrently admitted non-trauma patients [7]. The 2006 American College of Surgeons (ACS) Committee on Trauma (COT) Optimal Resources Document (ORD) focused on pre-hospital triage, stated that “it was generally agreed” a rate of 25 to 50% of non-major trauma patients taken to a trauma center (over triage) was acceptable to maintain a rate of major trauma patients taken to a non-trauma center (under triage) at less than 5%. In the 2014 ORD, stated “Most agree that an acceptable percentage of over triage is in the range of 25% to 35%,” prioritizing sensitivity over specificity and patient safety over the improper use of resources, whereas the under triage rate remained at 5% [8, 9]. In Europe, unlike in North America, pre-hospital care for severe trauma is usually provided by nurses and doctors, therefore, with the possibility of exploiting this expertise also in the triage procedure [10].

Multiple field-based trauma decision tools have attempted to standardize criteria for triage and ensure consistency of decision-making to minimize under- and over triage [11]. Despite these attempts, no consensus currently exists on the optimal triage tool. This review aims to evaluate the accuracy of triage tools for major trauma patients in the field.

## Methods

### Study design and setting

We conducted a systematic review to support the major trauma integrated management guideline panel of Italian National Institute of Health (Istituto Superiore di Sanità-ISS) [12] in formulating recommendations. Specifically, following the GRADE-ADOLOPMENT approach for guideline production [13] adopted by the ISS methodological manual [14], the panel members decided to apply a structured and systematic adaptation and updating process of the recommendation on the utilization of pre-hospital triage tools from NICE NG40 [15]. The clinical question addressed in this systematic review was: “Are the pre-hospital triage tools accurate in predicting adequate destination of severely injured patients to a trauma center (TC)?”

### Registered protocol

The SR followed the Preferred Reporting Items for Systematic Review and Meta-Analysis of Diagnostic Test Accuracy Studies (PRISMA-DTA) guideline [16, 17]. Study protocol has been stored at the following link: https://osf.io/846c2.

### Inclusion criteria

Test-treatment randomized controlled trials and/or observational studies were included if they met the following criteria: (1) *population*: children, young people, and adults experiencing trauma; (2) evaluation of the following *validated index triage tools*: modified Rapid Emergency Medicine Score (mREMS), the trauma score/revised trauma score (T/RTS), the current London “major trauma decision tool” (UK trauma tool), American College of Surgeons Committee on Trauma (ACS-COT) Field Decision Tool, Physiologic Severity Score (PSS), GAP (“Glasgow Coma Scale, Blood Pressure, Age”), MGAP (the previous plus “Mech of injury”); Vittel Triage criteria; new Trauma Team Activation (TTA); Northern French Alps Trauma System (TRENAU); New Trauma Score (NTS); Kampala Trauma Score (KTS); pre-hospital index (PHI); pre-hospital pediatric triage tools; (3) *reference standard test*: Injury Severity Score (ISS) higher than 15 as definition of major trauma, survival/mortality, ICU admission references can also be expressed as followed: number of patients identified as a major trauma at emergency department admission; in-hospital mortality at 4 weeks; mortality within 72 h after injury; admission to intensive care unit (ICU) within 72 h; number of patients transferred soon after arrival to a higher level of care (within 24 h); number of patients directly admitted from the emergency department to the operating room, Angio suite, or ICU (4) *Setting*: pre-hospital.

We excluded studies set in the North America, South America, and Asia due to the high incidence of penetrating trauma in these regions [18].

### Outcomes

The primary outcome of interest was the sensitivity and specificity of pre-hospital triage tools. Secondary outcomes were the percentages of under triage and over triage. Studies were required to have sensitivities and specificities value, a 2 × 2 table would be also adequate or to have provided enough information for the creation of a 2 × 2 table.

### Search strategy

We searched the following electronic databases: MEDLINE (PubMed), EMBASE (Elsevier, EMBASE.com), and Cochrane Central Register of Controlled Trials (CENTRAL) with language restriction (English, Italian, Spanish, French, German) using the search strategy outlined in the Supplement A of the high quality clinical guideline of the National Institute for Health and Clinical Excellence on major trauma [15]. We updated the search performed by NICE from 2015 up to November 2019. We checked the reference lists of all included studies and of any systematic reviews identified during the search process. We also searched gray literature such as Italian Regional registers (i.e., AREU, Azienda Regionale Emergenza Urgenza of Lombardia, Italy).

### Selection of studies and data extraction

Two independent authors screened titles and abstracts obtained by the search strategy (SG, GC). Each reviewer then independently assessed the full text of potentially relevant studies for inclusion. Any disagreement was solved by discussion with a third reviewer (OC). We adopted a standardized data collection form to extract the following information: study design, number, countries and settings, funding, duration of study, characteristics of participants, index test, reference test, types of outcomes, and length of follow-up. We contacted authors if the reported data were insufficient or unclear.

### Risk of bias assessment

Assessment of methodological quality of the included studies was evaluated using the Quality Assessment of Diagnostic Accuracy Studies version 2 (QUADAS-2) checklists [19]. Risk of bias and applicability in primary diagnostic accuracy studies in QUADAS-2 consists of 4 domains: patient selection, index test, reference standard, flow and timing.

### Data synthesis

Data synthesis was provided separately for adults and pediatric populations.

For diagnostic test accuracy studies, specific thresholds were defined and values above or below threshold—depending on different measures—were considered positive. The following measures were used for the analysis of the diagnostic test accuracy: area under the receiver operating characteristics (ROC) curve and, for different thresholds (if appropriate), sensitivity, and specificity. The threshold of a diagnostic test is defined as the value at which the test can be best differentiated between those with and without the target condition and it varies among studies. In triage tools, sensitivity (Sn) to detect major trauma was considered more important than specificity (SP) due to the consequences of a missed injury. A low sensitivity indicates that the test underestimates severity and a portion of major traumas are not recognized by the triage tool, thus causing under triage. A low specificity means that the test overestimates the severity of patients provoking over triage. Coupled forest plots of sensitivity and specificity with 95% CIs across studies (at various thresholds) were produced for each test, using RevMan [20]. In order to obtain this, 2 × 2 tables (the number of true positives, false positives, true negatives, and false negatives) were directly taken from the study (if given), derived from raw data, or calculated from the set of test accuracy statistics.

Diagnostic meta-analysis was conducted when 5 or more studies were available per threshold [15]. Test accuracy for the studies was pooled using the bivariate method modeled in STATA [21]. The bivariate method uses logistic regression on the true positives, true negatives, false positives, and false negatives reported in the studies. Overall sensitivity and specificity and confidence regions were plotted [22]. If studies evaluated the same triage tool, median sensitivity and specificity were reported, whenever possible.

AUC was also plotted on a graph for each diagnostic test. The AUC resumes the overall diagnostic accuracy across the full range of thresholds. The following criteria are used for evaluating AUC: ≤ 0.50, worse than chance; 0.51–0.60, very poor; 0.61–0.70, poor; 0.71–0.80, moderate; 0.81–0.90, good; 0.91–1.00, excellent or perfect test.

Finally, the net benefit was calculated for all models at different thresholds [23]. The net benefit represents the potential gain of using the prediction models under study for triage of injured patients compared with the effects of sending all patients to a major trauma center. Net benefit is defined as the proportion of true-positives—proportion of false-positives × weight. For example, a threshold of 0.2 means that a trauma center would accept four patients wrongly classified as having major trauma (false-positives) to identify one true major trauma (true-positive, defined as ISS over 15). The weight is defined as the odds of the threshold (maximum number of patients wrongly classified as having major trauma (false-positives) to correctly classify 1 patient with major trauma (true-positive)). For the threshold of 0.2, the weight is 4:1 [23].

Data were analyzed using RStudio software version 1.3.959 [24]. Heterogeneity or inconsistency among studies was visually inspected in the forest plots where there were similar thresholds.

### Certainty of the evidence

The GRADE approach was used, with five dimensions (risk of bias, consistency of effect, imprecision, indirectness, and publication bias) to assess the certainty of the body of the evidence [25]. The evidence was downgraded from “high quality” by one level if serious, or by two levels if very serious limitations are found for each of the five dimensions. We developed a “summary of findings” table presenting the certainty of the evidence, reasons for limitation, and main findings for the primary outcome in simple tabular format.

## Results

### Study selection

**Fig. 1:**
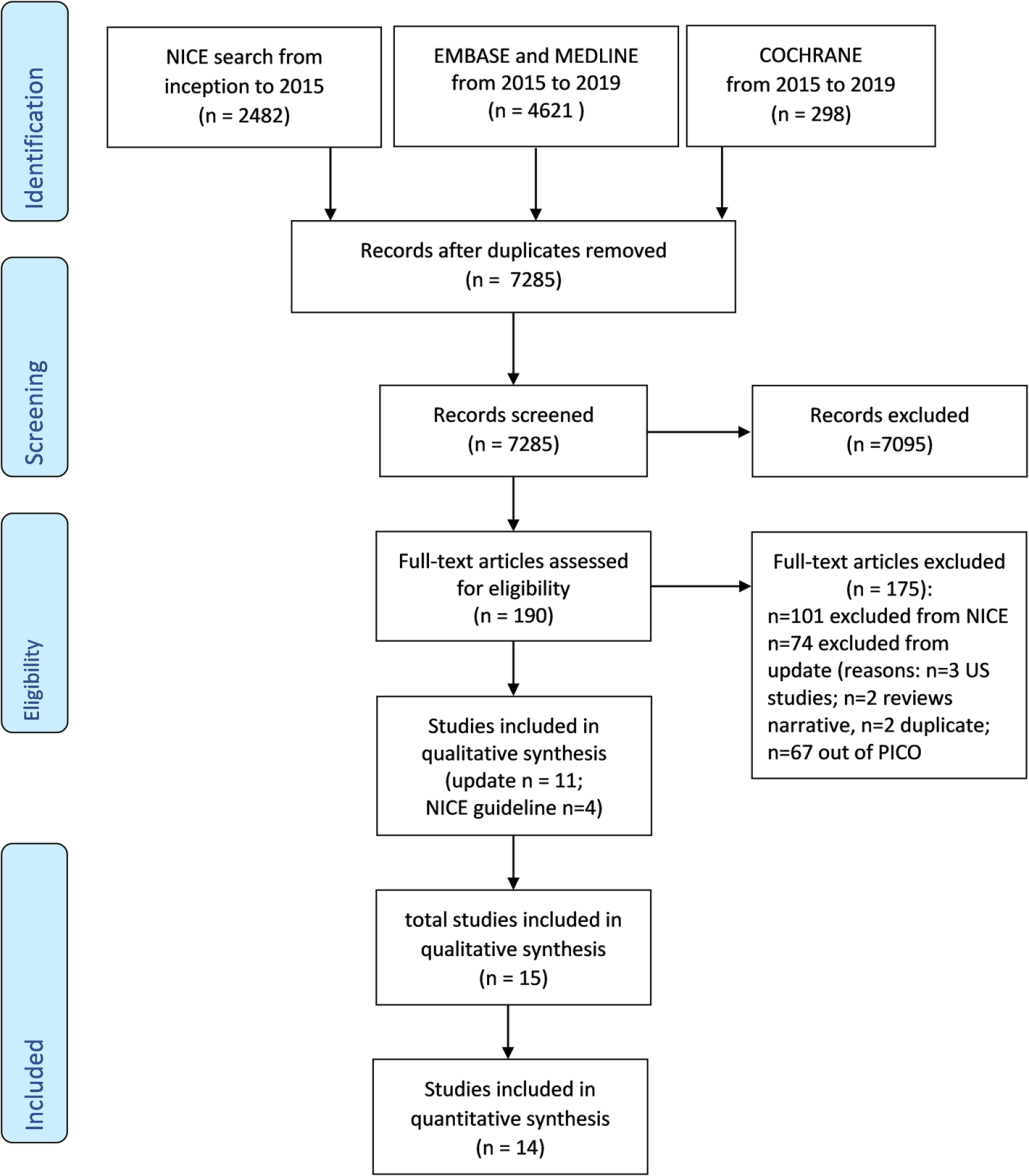
Study flow diagram

A total of 7285 records were screened. Reasons for exclusion were reported in Fig. 1. No additional studies from gray literature were found. From the updating search, 11 studies were included. Considering the 4 studies derived from the updated NICE guideline, a total of 15 studies were considered eligible for qualitative analysis and 14 studies for quantitative analysis (Fig. 1, flow diagram).

### Characteristics of included studies

None of the included studies were test-treatment RCTs allowing to establish a direct link between test and consequent management effect. Only observational studies were found. Three studies were prospectively collected whereas all other studies were retrospective. The whole number of recruited people was 210,285 of which 32,231 were children. The median number of participants included in the study was 1607 (IQR 1 076-3 344). Five studies were set in France, three in the UK (TARN network), three in the Netherlands, one in Australia, and one in multiple European countries (Spain, Denmark, Norway). Only five studies were funded. The duration of studies ranged from few months (1-5 months) up to 5 years. In 13 studies, the reference standard was the ISS > 15; in four, the mortality; in one, the ICU admission. For details, see Supplement B.

### Accuracy of pre-hospital triage tools in adults

**Fig. 2:**
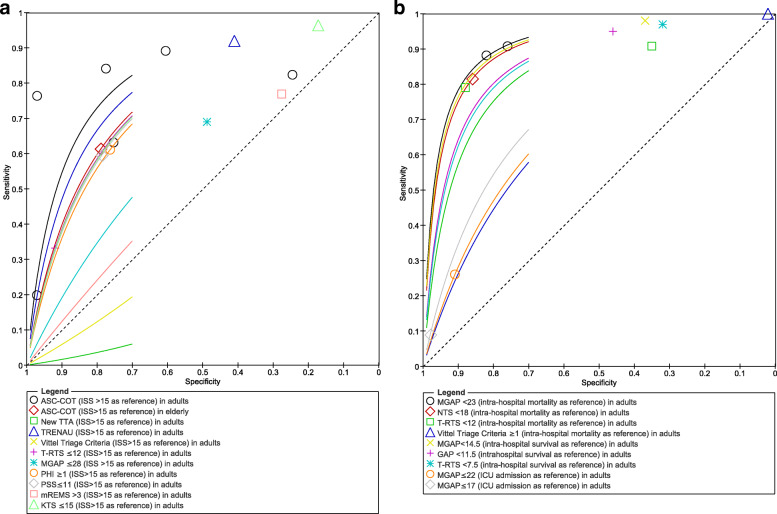
Summary ROC plot—accuracy trauma tools tests in adults: **a** (ISS > 15), **b** (mortality, survival, and ICU length stay)

A total of 13 studies reported data on triage tools accuracy [26–38]. Thirteen different tools were retrieved. Using ISS > 15 as reference, the cumulative evidence presented with the ACS-COT tool (6 studies in adults) showed a substantial heterogeneity in the accuracy evaluation (Supplement C - Figure 4) with Sn of 0.79 (median 95% CI, 0.73 to 0.83; low certainty of evidence) and Sp of 0.76 (median, 95% CI, 0.72 to 0.81; low certainty of evidence). Using mortality as reference, the cumulative evidence presented for the MGAP tool (2 studies in adults) showed a Sn of 0.90 (median, 95% CI, 0.82 to 0.94; moderate certainty of evidence) and Sp of 0.79 (median, 95% CI, 0.77 to 0.81; high certainty of evidence); the T-RTS tool (2 studies in adults) showed a cumulative evidence with a Sn of 0.85 (median, 95% CI, 0.77 to 0.91; moderate certainty of evidence); and a Sp of 0.61 (median, 95% CI, 0.59 to 0.64; high certainty of evidence). Figure 2 reports the summary ROC plot for all tools. Other data about accuracy of trauma tools in adults are reported in Supplement C (Figure 2a-b, forest plot).

One study [32] was included only in qualitative analysis since it investigated the performance of each category of the Vittel Criteria Algorithm (VCA) in predicting the risk of an ISS > 15, mortality within 30 days, or admission to Intensive Care Unit (*N* = 2 764): three algorithm categories were predictive of a major trauma patient (ISS > 15), physiological variables, pre-hospital resuscitation, and physical injuries, while kinetic elements were not. However, this study did not provide overall sn and sp values needed for quantitative analyses.

Under triage and over triage in adults were reported by 12 studies for 13 triage tools. Adopting ISS > 15 as reference test, the under triage ranged from 3.6 to 66.8%, while for over triage ranged from 3 to 87%. Considering mortality as reference test, the under triage ranged from 0 to 21%, while over triage ranged from 12 to 98%. Considering survival as a reference (1 study, 3 tools) the under triage data ranged from 2 to 5% while over triage from 54 to 68%. Finally, using admission to intensive care as reference test, the only tool tested (1 study, MGAP) offered very high values of under triage (74-91%) compared to negligible values of over triage (2-9%). Details about under triage and over triage for triage tools in adults are reported in Table 1. Predictive and negative values for adults are reported in Supplement C - Table 2.

**Table 1:** Under triage and over triage of triage trauma tools in adults

**Index test vs reference standard: ISS > 15**					
**Study ID**	**INDEX tool**	**All cases (n trauma patients)**	**ISS > 15**	**Under triage (%)**	**Over triage (%)**
Dinh 2012	ASC-COT	2664	285	37	25
Do 2014	ASC-COT	1696	182	24	3
Ocak 2009	ASC-COT	302	151	16	23
Bouzat 2015	ASC-COT	2572	1,185	17.6	76.6
Voskens 2018	Dutch field triage protocol (ACS-COT)	4950	436	21.6	30.6
Voskens 2018 (elderly > 65)	Dutch field triage protocol (ACS-COT)	1085	132	38.6	21.1
van Laarhoven 2014	Dutch field triage protocol (ACS-COT)	1607	221	10.9	39.5
Vinjevoll 2018	New trauma team activation criteria	998	127	-	87
Bouzat 2015	TRENAU	2572	1,185	8.5	58.8
Follin 2016	Vittel Triage Criteria	1160	417	-	64
Sewalt 2016	PHI ≤ 1 of 20	154,476	52,818	38.9	23.7
Sewalt 2016	T-RTS ≤ 11 of 12	154,476	52,818	66.8	8.1
Sewalt 2016	PSS ≤ 11 of 12	154,476	52,818	40.5	21.3
Sewalt 2016	MGAP ≤ 28 of 29	154,476	52,818	31	51.2
Sewalt 2016	mREMS > 3 of 26	154,476	52,818	23.1	72.4
Sewalt 2016	KTS ≤ 15 of 16	154,476	52,818	3.6	82.8
**Index test vs reference standard: in-hospital mortality**					
**Study ID**	**INDEX tool**	**All cases (n trauma patients)**	**Deaths**	**Under triage (%)**	**Over triage (%)**
Bouzat 2016	MGAP < 23	3260	186	12	18
Bouzat 2016	T-RTS < 12	3260	186	21	12
Cassignol 2019 a	T-RTS < 12	1001	76	9	65
Cassignol 2019 a	Vittel Triage Criteria ≥ 1	1001	76	0	98
Cassignol 2019 a	MGAP < 23	1001	76	9	24
Cassignol 2019 a	NTS (New Trauma Score) < 18	1001	76	18	14
**Index test** ***vs reference standard*: survival**					
**Study ID**	**INDEX tool**	**All cases (n trauma patients)**	**Survivals**	**Under triage (%)**	**Over triage (%)**
Llompart-Pou 2016	MGAP < 14.5	1361	1120	2	63
Llompart-Pou 2016	GAP < 11.5	1361	1120	5	54
Llompart-Pou 2016	T-RTS	1361	1120	3	68
**Index test** ***vs reference standard:*** **Intensive care unit (ICU) length of stay (LOS)**					
**Study ID**	**INDEX tool**	**All cases (n trauma patients)**	**ICU LOS**	**Under triage (%)**	**Over triage (%)**
Follin 2016	MGAP < 22	1160	475	74	9
Follin 2016	MGAP < 17	1160	475	91	2

Considering accuracy across all tools, the best trajectory curve seemed to be the one provided by the TRENAU tool when considering ISS > 15 as reference (total number, 2 572; Sn 0.92 and Sp 0.41; 1 study) and the NTS < 18 when in-hospital mortality was considered as a reference (total number, 1001; Sn, 0.82; Sp, 0.86; 1 study). See Supplement D for net clinical benefit curves.

### Accuracy of pre-hospital tools in children

**Fig. 3:**
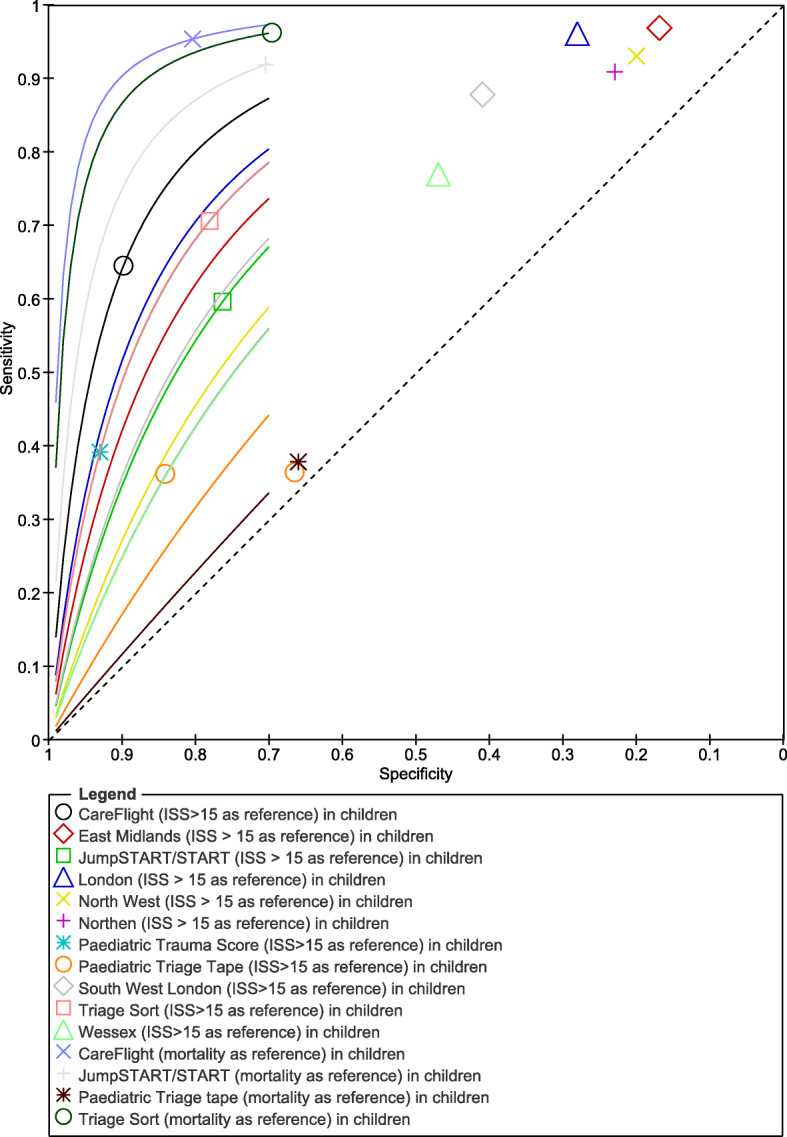
SROC plot—accuracy trauma tools test in children. Reference standards, ISS > 15 and mortality

A total of two studies reported data on triage tools accuracy in children [39, 40]. Eleven different tools were retrieved. Only one tool (Pediatric Triage Tape) was investigated by both studies. With ISS > 15 as reference, the cumulative evidence presented by Pediatric Triage Tape tool showed a median Sn of 0.36 (95% CI, 0.31 to 0.42; low certainty of evidence) and median Sp of 0.75 (95% CI, 0.72 to 0.78; low certainty of evidence). Figure 3 reports the summary ROC plot for all tools. All accuracy trauma tools tested in children are reported in Supplement C (Figure 5, Forest plot).

Considering ISS > 15 as reference, two out of 11 tools tested in children showed an undertriage lower than 5% (East Midland, 3% and London Triage, 4%), with an over triage of 83% and 72%, respectively. Considering mortality as reference, two out of the four tools tested in children showed under triage rates below 5% (CareFlight, 4.7% and Triage Sort, 3.8%) with an over triage of 19.6% and 30.4%, respectively. Under triage and over triage of trauma tools in children are reported in Supplement C- Table 1. Predictive and negative values are reported in Supplement C- Table 3.

All the instruments considered were analyzed by only two studies with different sample size: *n* = 31292 and *n* = 701 for Price and Cheung, respectively. Considering all the accuracy measurements, using ISS > 15 as reference, the CareFlight instrument had the best net clinical benefit curve, as also demonstrated by ROC values, with the highest curve among all tools, while the Pediatric Triage Tape instrument had the net worst clinical benefit, having the lowest ROC curve. With mortality as reference, according to the ROC curve, the CareFlight tool showed higher Sn and Sp, followed by the Triage Sort, JumpSTART/START, and Pediatric Triage Tape tools. See Supplement D for net clinical benefit curves.

### Risk of bias assessment and certainty of the evidence

Generally unclear risk of bias was present across studies. See Supplement E.

In adults, for the ACS-COT instrument (ISS > 15 as a reference test) the certainty of the evidence of the tests for Sn and Sp were low (Supplement F - Table 1). For the MGAP instrument (mortality as a reference test, Supplement F – Table 2) and for the T-RTS (mortality as a reference test, Supplement F – Table 3), the Sn was of moderate certainty of the evidence and the Sp of high certainty of the evidence. The diagnostic accuracy of the remaining tools for adults was always of moderate (NTS and New Trauma team activation criteria) or high certainty of the evidence (TRENAU and Vittel Triage Criteria) (Supplement F – Table 5).

In children, the certainty of the evidence of the tests for the Sn and Sp of the Pediatric Triage Tape instrument (ISS > 15 as reference) was low (Supplement F – Table 4), and moderate or high for the remaining tools (Supplement F – Table 6). For details, see Supplement F.

## Discussion

In this systematic review, we found high variability in Sn and Sp among all currently available studies on pre-hospital trauma triage tools with cumulative certainty of evidence ranging from moderate-high (MGAP, 2 studies and T-RTS, 2 studies) to low (ACS-COT, 2 studies) in adults. Certainty of evidence was high for the TRENAU and Vittel Triage Criteria tools, and moderate for New Trauma team activation criteria and NTS in adults, all represented by single studies.

As for pediatric trauma triage tools, the cumulative certainty of evidence was low for Pediatric Triage Tape, (2 studies) but moderate (London, East Midland, North West, South West London, Wessex) to high (Care Flight, JumpSTART/START, Triage short) for tools reported in single studies. In terms of accuracy, net benefit curves, under triage and over triage, no definite conclusion can be made on the use of pre-hospital pediatric triage tools and most of the methods which have been evaluated are mainly applied in the setting of multiple events or maxi-emergencies.

Our results confirmed the wide range of under triage and over triage in adults found in a recent systematic review [1] where a comparative analysis was not performed but contrasting results of triage protocols were underlined. The authors reported a general poor methodological quality of included studies, but certainty of evidence was not explored. As in our review, none of the trauma systems included had an under triage rate below 5%, combined with an over triage rate below 35%, as recommended by the ACS-COT [8].

Our results were also in agreement with current scientific literature for children’s triage tools: a previous systematic review on accuracy [41] found that none of the investigated field triage tools complied with the international standard of 95% or greater sensitivity to prevent under triage and its potential life-threatening consequences. Indeed, all tools reached recommended standards for over triage, but the majority favored under triage [42].

Pre-hospital assessment of the trauma patient is challenging. The evaluation of the scene is often imprecise: some indicators of high energy mechanism, such as high speed, are reported by witnesses in an approximate way, while others such as vehicle roll-over, entrapment with compartment intrusion, and comorbidities are not statistically associated with severe injuries and can be the cause of an elevated over triage [34]. Moreover, the development of high technology safety devices for drivers or passengers of vehicles has substantially reduced the severity of injuries following road-related accidents and many mechanisms highlighted in historical studies should be reconsidered. On the other hand, an alteration in patient physiology (consciousness, breathing and circulation) or the anatomic evidence of a major injury are generally associated with lower rates of over triage [43, 44]*.*

Pre-hospital care is directed differently worldwide. While in North-American countries, health care on the scene is given by paramedics; in Europe, pre-hospital crews are mainly represented by doctors and nurses who may be able to obtain a more accurate assessment of the patient’s clinical status and administer immediate treatment on the scene [10](https://www.euro.who.int/__data/assets/pdf_file/0016/114406/E92038.pdf). In our analysis, the TRENAU system was the triage tool with the best trajectory curve and performance with ISS > 15 as reference. This method, introduced in France in 2008, describes three codes of severity: (A) unstable despite resuscitation, (B) unstable but responsive to on-scene therapeutic intervention (tracheal intubation, infusions), or anatomy of major injury, (C) stable with high-kinetic mechanism or co-morbidities [29]. While A and B patients should be conveyed to the highest level of care, C patients can be admitted to lower-level facility. Under triage was about 9%; it was reduced to 1% when the same criteria were applied in an urban context, outlining that the pre-hospital medical evaluation enhances the quality of triage [45]*.* The over triage higher than 55% could be probably reduced if all triage code C patients were admitted to a level 2 trauma center.

With mortality as reference, the NTS was associated with a good combination of sensitivity and specificity, respectively 82% and 86%. NTS is based on easily available physiological parameters, systolic blood pressure, GCS, and SpO2. Many studies showed that respiratory rate measurement on the field or ED is inaccurate, due to patient conditions, emotional factors, loud noises, even if an electronic device is used [46, 47]. In addition, respiratory rate is often not recorded in patients who are intubated on the scene. SpO2 is an objective and unequivocal parameter largely used as simple tool to assess peripheral oxygenation. A cut-off value of NTS 18, used for centralization to the trauma center, showed a better discrimination than the RTS (AUC 0.935 vs 0.917) and the specificity at a fixed sensitivity of 95% was over 82%, which outperforms RTS [32, 48]. Based on our results, the use of TRENAU together with NTS seems to be a good option for the pre-hospital triage in an inclusive system with doctors and nurses on the scene. This combination maximizes the role of high-level pre-hospital care, with a professional evaluation on scene by experienced personnel. In the inclusive model, the system takes care of trauma patients of the area suffering from any type of traumatic injury. The role of a pre-hospital emergency medical system is of paramount importance, as it must be able to identify major trauma patients on the scene and ensure the patient’s admission in the shortest time to the hospital capable of providing a definitive care of injuries.

## Limitations

This is the first systematic review that includes the certainty of the evidence with the GRADE approach for studies in the pre-hospital setting, assessing the accuracy of pre-hospital triage tools. This method should increase the external validity and clinical relevance of the findings. However, some limitations must be acknowledged. First, we restricted our inclusion criteria only to European pre-hospital settings since prevalence rates of blunt and penetrating trauma are slightly different from other continents; however, this might led to geographical bias and selection bias limiting generalizability and interpretability of our results [49]. Indeed, North America had the highest percentage of penetrating trauma (16%), followed by Asia (15%), South America (13%), and then Europe and Oceania (both 4%) [18]. Thus, it was considered inappropriate to group these regions’ data since the existing variability in trauma incidence and health care system organization across countries. In fact, the out-of-hospital use of physicians in a majority of the reviewed countries is not a universal standard and can greatly alter the care that can be given in the out-of-hospital setting. Second, we did not find randomized controlled trials (RCTs) comparing test-treatment interventions: RCTs are able to provide good data given their rigorous methods for evaluating the effectiveness of diagnostic tests [50]. It can be argued whether performing a large randomized controlled trial is unethical in life threating situations [51]. A cluster randomized controlled trial by healthcare hospital and territories may be a valuable tool in emergency medicine research to compare pre-hospital triage tools in terms of sensitivity, specificity, under triage, and over triage [52]. The stepped-wedge designs allow for within and between cluster comparisons [53, 54]. However, difficulties may be encountered when recruiting participants into emergency setting trials: the assessment of external validity and applicability of trial results is therefore essential [55]. Moreover, the isolated performance of a diagnostic test is indeed difficult to interpret and could differ according to the context depending on the prevalence of the condition [56]. Third, the included studies were of unclear quality and with a retrospective design, thereby limiting the conclusions that can be drawn. Forth, the different triage tools had non-comparable reference standards as surrogate markers of the need of a specialized trauma care: most studies, we included the use ISS > 15 or mortality as reference, both of which are limited in their ability to predict the need for trauma resources making interpretation of results even more challenging. One need in triage research is the use of established and consistent outcomes.

## Conclusion

In adults, the certainty of evidence was high in TRENAU and Vittel Triage Criteria whereas in the pediatric field a large variability prevented any firm conclusion in European and Australian pre-hospital care that operates within a leveled trauma care system. In our systematic review, we found high variability in terms of sensitivity and specificity in all currently available studies on pre-hospital trauma triage tools. Furthermore, several tools are compared with several reference standards. In an inclusive Health System, which considers the whole spectrum of trauma, from minor to more severely injured, the adoption of an accurate pre-hospital triage tool may help to allocate trauma patients according to hospital resources. It is of paramount importance to match the right patient with the right hospital to maximize the healthcare and to minimize costs.

## Supplementary Information

**Additional file 1: Supplement A.** Search strategies. **Supplement B.** Characteristics of included studies. **Supplement C.** Accuracy data of pre-hospital tools. **Supplement D.** Net clinical benefit curves. **Supplement E.** Quality assessment QUADAS 2. **Supplement F.** Summary of findings tables.

## Data Availability

All data generated or analyzed during this study are included in this published article [and its additional files, https://osf.io/846c2].

## Abbreviations

ACS-COT: American College of Surgeons Committee on Trauma
AUC: Area under the curve
GAP: Glasgow Coma Scale, Blood pressure, Age
GRADE: Grading of Recommendations Assessment, Development and Evaluation
ISS: Injury Severity Score
KTS: Kampala Trauma Score
MGAP: Glasgow Coma Scale, Blood pressure, Age plus “Mech of injury”
Mrems: Modified Rapid Emergency Medicine Score (mREMS)
NTS: New Trauma Score
PHI: Pre-hospital index
PSS: Field Decision Tool, Physiologic Severity Score
QUADAS-2: Quality Assessment of Diagnostic Accuracy Studies version 2 checklists
RCT: Randomized controlled trials
ROC: Receiver operating characteristics
Sn: Sensitivity
Sp: Specificity
T/RTS: Trauma score/Revised Trauma Score (T/RTS)
TRENAU: Northern French Alps Trauma System
TTA: New Trauma Team Activation
