# Supplementary material for: Accuracy of pre-hospital triage tools for major trauma: a systematic review with meta-analysis and net clinical benefit
Source: World J Emerg Surg. 2021 Jun 10;16:31. doi: 10.1186/s13017-021-00372-1 (PMC8193906; doi:10.1186/s13017-021-00372-1)
Supplement: Supplementary file 1 — Additional file 1: Supplement A. Search strategies. Supplement B. Characteristics of included studies. Supplement C. Accuracy data of pre-hospital tools. Supplement D. Net clinical benefit curves. Supplement E. Quality assessment QUADAS 2. Supplement F. Summary of findings tables. [file 13017_2021_372_MOESM1_ESM.pdf]

Supplementary Materials

Supplement A. Search strategies 2
Supplement B. Characteristics of included studies..12
Supplement C. Accuracy data of pre-hospital tools 27
Supplement D. Net clinical benefit curves 38
Supplement E. Quality assessment QUADAS 2 51
Supplement F. Summary of findings tables..52

Supplement A. Search strategies

Standard major trauma population

Medline search

1.	(trauma* or polytrauma*).ti,ab.
2.	((serious* or severe* or major or life threaten*) adj3 (accident* or injur* or fall*)).ti,ab.
3.	multiple trauma/
4.	wounds, gunshot/ or wounds, stab/ or accidents, traffic/ or accidental falls/ or blast injuries/ or accidents, aviation/
5.	((motor* or motorbike* or vehicle* or road or traffic or car or cars or cycling or bicycle* or automobile* or bike* or head on or pile up) adj3 (accident* or crash* or collision* or smash*)).ti,ab.
6.	(mvas or mva or rtas or rta).ti,ab.
7.	(stabbed or stabbing or stab or gunshot* or gun or gunfire or firearm$ or bullet* or knife* or knives or dagger).ti,ab.
8.	or/1-7

Embase search terms

1.	(trauma* or polytrauma*).ti,ab.
2.	((serious* or severe* or major or life threaten*) adj3 (accident* or injur* or fall*)).ti,ab.
3.	multiple trauma/
4.	gunshot injury/ or stab wound/ or traffic accident/ or falling/ or blast injury/ or aircraft accident/
5.	((motor* or motorbike* or vehicle* or road or traffic or car or cars or cycling or bicycle* or automobile* or bike* or head on or pile up) adj3 (accident* or crash* or collision* or smash*)).ti,ab.
6.	(mvas or mva or rtas or rta).ti,ab.
7.	(stabbed or stabbing or stab or gunshot* or gun or gunfire or firearm$ or bullet* or knife* or knives or dagger).ti,ab.
8.	or/1-7

Cochrane search terms

#1.	MeSH descriptor: [multiple trauma] this term only
#2.	(trauma* or polytrauma*):ti
#3.	((serious* or severe* or major) near/3 (accident* or injur* or fall*)):ti
#4.	MeSH descriptor: [wounds, gunshot] this term only

#5.	MeSH descriptor: [wounds, stab] this term only
#6.	MeSH descriptor: [accidents, traffic] this term only
#7.	MeSH descriptor: [accidental falls] this term only
#8.	MeSH descriptor: [blast injuries] this term only
#9.	MeSH descriptor: [accidents, aviation] this term only
#10.	((motor* or motorbike* or vehicle* or road or traffic or car or cars or cycling or bicycle* or automobile* or bike*) near/3 (accident* or crash* or collision* or smash*)):ti
#11.	(mvas or mva or rtas or rta):ti
#12.	(stabbed or stabbing or stab or gunshot or gun or gunfire or firearm* or bullet or knife* or knives or dagger or shot):ti
#13.	{or #1-#12}

Expanded trauma population

The following terms were **combined** with F.2.1 using the **OR Boolean operator**

Medline search

1.	exp emergency service, hospital/
2.	emergency medical services/
3.	((emergency or emergencies) adj2 (department* or dept* or unit* or room* or ward* or service* or team* or hospital* or medic* or centre* or center*)).ti,ab.
4.	"accident and emergency".ti,ab.
5.	a&e.ti,ab.
6.	ed.ti,ab.
7.	walk-in centre*.ti,ab.
8.	minor injuries unit*.ti,ab.
9.	exp fractures, bone/
10.	fracture*.ti,ab.
11.	exp spinal injuries/
12.	exp spinal cord injuries/
13.	spinal cord compression/
14.	exp neck injuries/
15.	((spine or spinal or vertebr* or neck or cervical or lumbar or sacral or thoracic or cord or whiplash) adj2 (injur* or damag* or trauma* or fracture* or compress* or contus* or lacerat* or transect* or lesion*)).ti,ab.

16.	(central cord syndrome or central spinal cord syndrome).ti,ab.
17.	(conus medullaris syndrome* or cauda equina syndrome*).ti,ab.
18.	or/1-17

Embase search terms

1.	exp spine injury/
2.	neck injury/ or whiplash injury/
3.	exp spinal cord injury/
4.	((spine or spinal or vertebr* or neck or cervical or lumbar or sacral or thoracic or cord or whiplash) adj2 (injur* or damag* or trauma* or fracture* or compress* or contus* or lacerat* or transect* or lesion*)).ti,ab.
5.	(central cord syndrome or central spinal cord syndrome).ti,ab.
6.	(conus medullaris syndrome* or cauda equina syndrome*).ti,ab.
7.	emergency health service/
8.	((emergency or emergencies) adj2 (department* or dept* or unit* or room* or ward* or service* or team* or hospital* or medic* or centre* or center*)).ti,ab.
9.	ed.ti,ab.
10.	"accident and emergency".ti,ab.
11.	a&e.ti,ab.
12.	walk-in centre*.ti,ab.
13.	minor injuries unit*.ti,ab.
14.	fracture/
15.	fracture*.ti,ab.
16.	or/1-15

Cochrane search terms

#1.	MeSH descriptor: [emergency service, hospital] explode all trees
#2.	MeSH descriptor: [emergency medical services] this term only
#3.	((emergency or emergencies) near/2 (department* or dept* or unit* or room* or ward* or service* or team* or hospital* or medic* or centre* or center*)):ti,ab
#4.	ed:ti,ab
#5.	"accident and emergency":ti,ab
#6.	a&e:ti,ab
#7.	walk-in centre*:ti,ab
#8.	minor injuries unit*:ti,ab

#9.	MeSH descriptor: [fractures, bone] explode all trees
#10.	fracture*:ti,ab
#11.	MeSH descriptor: [spinal injuries] explode all trees
#12.	MeSH descriptor: [spinal cord injuries] explode all trees
#13.	MeSH descriptor: [spinal cord compression] this term only
#14.	MeSH descriptor: [neck injuries] explode all trees
#15.	((spine or spinal or vertebr* or neck or cervical or lumbar or sacral or thoracic or cord or whiplash) near/2 (injur* or damag* or trauma* or fracture* or compress* or contus* or lacerat* or transect* or lesion*)):ti,ab,kw
#16.	(central cord syndrome or central spinal cord syndrome):ti,ab,kw
#17.	(conus medullaris syndrome or cauda equina syndrome):ti,ab,kw
#18.	{or #1-#17}

Systematic review (SR) search terms

Medline search terms

1.	meta-analysis/
2.	meta-analysis as topic/
3.	(meta analy* or metanaly* or metaanaly*).ti,ab.
4.	((systematic* or evidence*) adj3 (review* or overview*)).ti,ab.
5.	(reference list* or bibliograph* or hand search* or manual search* or relevant journals).ab.
6.	(search strategy or search criteria or systematic search or study selection or data extraction).ab.
7.	(search* adj4 literature).ab.
8.	(medline or pubmed or cochrane or embase or psychlit or psyclit or psychinfo or psycinfo or cinahl or science citation index or bids or cancerlit).ab.
9.	cochrane.jw.
10.	((multiple treatment* or indirect or mixed) adj2 comparison*).ti,ab.
11.	or/1-10

Embase search terms

1.	systematic review/
2.	meta-analysis/
3.	(meta analy* or metanaly* or metaanaly*).ti,ab.
4.	((systematic or evidence) adj3 (review* or overview*)).ti,ab.

5.	(reference list* or bibliograph* or hand search* or manual search* or relevant journals).ab.
6.	(search strategy or search criteria or systematic search or study selection or data extraction).ab.
7.	(search* adj4 literature).ab.
8.	(medline or pubmed or cochrane or embase or psychlit or psyclit or psychinfo or psycinfo or cinahl or science citation index or bids or cancerlit).ab.
9.	((pool* or combined) adj2 (data or trials or studies or results)).ab.
10.	cochrane.jw.
11.	((multiple treatment* or indirect or mixed) adj2 comparison*).ti,ab.
12.	or/1-11

Randomized controlled trials (RCTs) search terms

Medline search terms

1.	randomized controlled trial.pt.
2.	controlled clinical trial.pt.
3.	randomi#ed.ab.
4.	placebo.ab.
5.	randomly.ab.
6.	clinical trials as topic.sh.
7.	trial.ti.
8.	or/1-7

Embase search terms

1.	random*.ti,ab.
2.	factorial*.ti,ab.
3.	(crossover* or cross over*).ti,ab.
4.	((doubl* or singl*) adj blind*).ti,ab.
5.	(assign* or allocat* or volunteer* or placebo*).ti,ab.
6.	crossover procedure/
7.	double blind procedure/
8.	single blind procedure/
9.	randomized controlled trial/

10.	or/1-9

Observational studies (OBS) search terms

Medline search terms

1.	epidemiologic studies/
2.	exp case control studies/
3.	exp cohort studies/
4.	cross-sectional studies/
5.	case control.ti,ab.
6.	(cohort adj (study or studies or analys*)).ti,ab.
7.	((follow up or observational or uncontrolled or non randomi#ed or nonrandomi#ed or epidemiologic*) adj (study or studies)).ti,ab.
8.	((longitudinal or retrospective or prospective or cross sectional) and (study or studies or review or analys* or cohort*)).ti,ab.
9.	or/1-8

Embase search terms

1.	clinical study/
2.	exp case control study/
3.	family study/
4.	longitudinal study/
5.	retrospective study/
6.	prospective study/
7.	cross-sectional study/
8.	cohort analysis/
9.	follow-up/
10.	cohort*.ti,ab.
11.	9 and 10
12.	case control.ti,ab.
13.	(cohort adj (study or studies or analys*)).ti,ab.
14.	((follow up or observational or uncontrolled or non randomi#ed or nonrandomi#ed or epidemiologic*) adj (study or studies)).ti,ab.
15.	((longitudinal or retrospective or prospective or cross sectional) and (study or studies or review or analysis* or cohort*)).ti,ab.

16.	or/1-8,11-15

Excluded study designs and publication types

The following study designs and publication types were removed from retrieved results using **the NOT** operator.

Medline search terms

1.	letter/
2.	editorial/
3.	news/
4.	exp historical article/
5.	anecdotes as topic/
6.	comment/
7.	case report/
8.	(letter or comment*).ti.
9.	or/1-8
10.	randomized controlled trial/ or random*.ti,ab.
11.	9 not 10
12.	animals/ not humans/
13.	exp animals, laboratory/
14.	exp animal experimentation/
15.	exp models, animal/
16.	exp rodentia/
17.	(rat or rats or mouse or mice).ti.
18.	or/11-17

Embase search terms

1.	letter.pt. or letter/
2.	note.pt.
3.	editorial.pt.
4.	case report/ or case study/
5.	(letter or comment*).ti.
6.	or/1-5

7.	randomized controlled trial/ or random*.ti,ab.
8.	6 not 7
9.	animal/ not human/
10.	nonhuman/
11.	exp animal experiment/
12.	exp experimental animal/
13.	animal model/
14.	exp rodent/
15.	(rat or rats or mouse or mice).ti.
16.	or/8-15

Pre-hospital alert and triage tools

Medline search terms – search A

1.	emergency medical services/ or triage/ or transportation/ or air ambulances/ or ambulances/ or "transportation of patients"/ or ambulance diversion/
2.	(prehospital* or pre hospital* or roadside* or road side* or triage or triaging).ti,ab.
3.	((accident* or trauma) adj2 (site* or scene* or location*)).ti,ab.
4.	((outside or out) adj2 hospital).ti,ab.
5.	(emergency adj2 (service* or staff or personnel)).ti,ab.
6.	(ambulance* or helicopter* or paramedic* or emergency medic* or emergency service* or emergency care or first respon*).ti,ab.
7.	or/1-6
8.	((trauma or triage or injur*) adj2 (scale* or tool* or score* or scoring or index*)).ti,ab.
9.	((risk or predict*) adj2 (scale* or tool* or score* or scoring or index*)).ti,ab.
10.	(decision adj2 (technique* or system*)).ti,ab.
11.	((decision or prediction) adj2 (tool* or rule*)).ti,ab.
12.	decision support techniques/
13.	trauma severity indices/ or abbreviated injury scale/ or glasgow coma scale/ or injury severity score/
14.	(london adj3 prog*).ti,ab.
15.	or/8-14
16.	7 and 15

Embase search terms – search A

1.	*emergency health service/
2.	*emergency care/ or *rescue personnel/ or *emergency patient/ or *emergency treatment/
3.	*ambulance/ or *ambulance diversion/ or *ambulance transportation/
4.	*patient transport/
5.	*air medical transport/
6.	*paramedical personnel/
7.	(prehospital* or pre hospital* or roadside* or road side* or triage or triaging).ti,ab.
8.	((accident* or trauma) adj2 (site* or scene* or location*)).ti,ab.
9.	((outside or out) adj2 hospital).ti,ab.
10.	(emergency adj2 (service* or staff or personnel)).ti,ab.
11.	(ambulance* or helicopter* or paramedic* or emergency medic* or emergency service* or emergency care or first respon*).ti,ab.
12.	or/1-11
13.	((trauma or triage or injur*) adj2 (scale* or tool* or score* or scoring or index*)).ti,ab.
14.	((risk or predict*) adj2 (scale* or tool* or score* or scoring or index*)).ti,ab.
15.	(decision adj2 (technique* or system*)).ti,ab.
16.	((decision or prediction) adj2 (tool* or rule*)).ti,ab.
17.	*decision support system/
18.	*injury scale/ or *glasgow coma scale/
19.	(london adj3 prog*).ti,ab.
20.	or/13-19
21.	12 and 20

Cochrane search terms – search A

#1.	MeSH descriptor: [emergency medical services] this term only
#2.	MeSH descriptor: [triage] this term only
#3.	MeSH descriptor: [transportation] this term only
#4.	MeSH descriptor: [air ambulances] this term only
#5.	MeSH descriptor: [ambulances] this term only
#6.	MeSH descriptor: [transportation of patients] this term only

#7.	MeSH descriptor: [ambulance diversion] this term only
#8.	(prehospital* or pre hospital* or roadside* or road side* or triage or triaging):ti,ab
#9.	((accident* or trauma) near/2 (site* or scene* or location*)):ti,ab
#10.	((outside or out) near/2 hospital):ti,ab
#11.	(emergency near/2 (service* or staff or personnel)):ti,ab
#12.	(ambulance* or helicopter* or paramedic* or emergency medic* or emergency service* or emergency care or first respon*):ti,ab
#13.	{or #1-#12}
#14.	((trauma or triage or injur*) near/2 (scale* or tool* or score* or scoring or index*)):ti,ab
#15.	((risk or predict*) near/2 (scale* or tool* or score* or scoring or index*)):ti,ab
#16.	(decision near/2 (technique* or system*)):ti,ab
#17.	((decision or prediction) near/2 (tool* or rule*)):ti,ab
#18.	MeSH descriptor: [decision support techniques] this term only
#19.	MeSH descriptor: [trauma severity indices] this term only
#20.	MeSH descriptor: [abbreviated injury scale] this term only
#21.	MeSH descriptor: [glasgow coma scale] this term only
#22.	MeSH descriptor: [injury severity score] this term only
#23.	london near/3 prog*:ti,ab
#24.	{or #14-#23}
#25.	#13 and #24

Supplement B. Characteristics of included studies

Cheung 2013

**Study**	**Cheung 2013**
Study type	Retrospective diagnostic cohort study (Trauma Registry)
Number of studies (number of participants	701
Countries and Settings	TARN registered hospitals; UK
Funding	None reported
Duration of study	5 years
Age, gender, ethnicity	(M: F) 2:1; Age: Not reported; Ethnicity: Not reported
Patient characteristics	People aged below 16 sustaining injury or trauma and admitted to a receiving unit direct from the scene of the incident.
Index test	UK Trauma Tools: East Midlands, London, North West, Northern, South West London, Wessex, Pediatric Trauma Score
Reference standard	Later clinical confirmation of Major Trauma: ISS >15

Dinh 2012

**Study**	**Dinh 2012**
Study type	Retrospective observational study (Trauma Registry)
Number of studies (number of participants	2.664
Countries and Settings	Sydney (urban city) Australia, Pre-hospital (Major Trauma Centre)
Funding	None reported
Duration of study	1 year
Age, gender, ethnicity	Non Major Trauma (non-MT): (M:F) 1:1; (Mean Age, SD) 57 (24); Gender: Not reported Major Trauma (MT): (M:F) 3:1; (Mean Age, SD) 42 (19); Gender: Not reported
Patient characteristics	All adult (>15) years old patients who were transported directly by the Ambulance Service of New South Wales (ASNSW) because of injury
Index test	ACS-SCOT: 2006 Triage rule
Reference standard	Later clinical confirmation of Major Trauma: Death<br>Later clinical confirmation of Major Trauma: ISS >15

Do 2014

**Study**	**Do 2014**
Study type	Retrospective observational study (Trauma Registry)
Number of studies (number of participants	1934
Countries and Settings	Denmark; Trauma Network - Tertiary hospitals and level 1 trauma centres
Funding	TrygFonden (Private Philanthropy)
Duration of study	1 and 5 months
Age, gender, ethnicity	Adult Population: (M: F) 2:1; (Mean Age, Range) 36 (22-51); Ethnicity: Not reported<br>Paediatric Population: (M:F) 1:1; (Mean Age, Range) 10 (6-13); Ethnicity: Not reported
Patient characteristics	All trauma patients aged 79 or less, with a minimum driving distance of 30 minutes to the regional TC, including self attendees.
Index test	ACS-SCOT: 2006 Triage rule (derivative)
Reference standard	Later clinical confirmation of Major Trauma: ISS>15

Ocak 2009

**Study**	**Ocak 2009**
Study type	Retrospective observational study (Trauma Registry)
Number of studies (number of participants	302
Countries and Settings	10 trauma centres (3 Level 1 centres) - Holland
Funding	None reported.
Duration of study	1 year
Age, gender, ethnicity	Non Major Trauma (non-MT): (M:F) 1:1; (Mean Age, SD) 59.7 (23.3); Gender: Not reported Major Trauma (MT): (M:F) 2:1; (Mean Age, SD) 48.4 (23.7); Gender: Not reported
Patient characteristics	Adult trauma patients who were transported by ambulance from the accident scene
Index test	ACS-SCOT: 2006 Triage rule
Reference standard	Later clinical confirmation of Major Trauma: ISS>15.

Follin 2016

**Study_ID 4**	**Follin 2016**
Study type	Prospective, observational study
Number of studies/participants	1.160
Countries and Settings	The study was performed in an 800-bed specialized Trauma Center (Hospital Europe´en Georges Pompidou) in Paris, France. Prehospital triage was performed by a physician-staffed prehospital EMS.
Funding	The work was supported only by institutional funding.
Duration of study	3-year study period
Age, gender, ethnicity	(M: F) 2:1; Age (mean, range): 35, 25-49; Ethnicity: Not reported
Patient characteristics	
Index test	Vittel Triage Criteria
Reference standard	Later clinical confirmation of Major Trauma: ISS >15

Voskens 2018

**Study _ID6**	**Voskens 2018**
Study type	Retrospective observational study
Number of studies/participants	4950 patients
Countries and Settings	10 hospitals Central Netherlands (9 level II and III hospitals and 1 level I trauma center) Trauma Registry
Funding	Not reported
Duration of study	Three years (from 2012 to 2014)
Age, gender, ethnicity	Adult Population: (M: F) 1:1; male 58.3%; median age 45 (22-51); Ethnicity: Not reported
Patient characteristics	All trauma patients 16 years and older transported by EMS professionals with the highest priority. Patients transported to a hospital outside Central Netherlands and patients transported by helicopter were excluded. They were also excluded if insufficient data were available in the receiving hospital to properly calculate the Injury Severity Score (ISS).
Index test	Dutch field triage protocol based on ACS-SCOT (2006 Triage rule)
Reference standard	Later clinical confirmation of Major Trauma: ISS >16

Price 2016

**Study_ID7**	**Price 2016**
Study type	Retrospective observational cohort study
Number of studies/participants	31.292 PEDIATRIC patients aged less than 16 years who sustained a traumatic injury.
Countries and Settings	Data were obtained from the UK Trauma Audit and Research Network (TARN) database
Funding	This work was supported by a grant from the Department of Health Emergency Department. The funder had no role in the design, analysis, interpretation of the results, or the writing of the manuscript.
Duration of study	August 2009
Age, gender, ethnicity	(M: F) 2:1; Age (mean± SD): 7.9 ± 4.9; Ethnicity: Not reported
Patient characteristics	Patients aged less than 16 years, respiratory rate (breaths per minute), systolic blood pressure (mmHg), cardiac arrest (yes/no), intubated (yes/no), age (years), capillary refill time (>2/<2 s), heart rate (beats per minute), Glasgow Coma Scale (GCS) score and Injury Severity Score (ISS)
Index test	JumpSTART, START, CareFlight, Paediatric Triage Tape/Sieve and Triage Sort
Reference standard	Later clinical confirmation of Major Trauma: ISS >15 Later clinical confirmation of Major Trauma: Mortality (time point not reported)

Vinjevoll 2018

**Study_ID8**	**Vinjevoll 2018**
Study type	Multi-center observational cohort study with retrospective data analysis
Number of studies/participants	998 were eligible for triage criteria analysis
Countries and Settings	Central Norway is one of four major health trusts in Norway. It covers an area of 56.385 km^2 and a total population of 680.110. St. Olav's University Hospital is the major trauma centre (MTC) and has formal responsibility for the regional trauma organization
Funding	The authors received no external funding
Duration of study	1 year, between 01.01.2015 to 31.12.2015
Age, gender, ethnicity	(M: F) 2:1; Age (median, range): 35, 20-58; Ethnicity: Not reported
Patient characteristics	Deaths prior to hospital arrival, patients without TTA and those transferred from other hospitals more than 24 h after injury were excluded.
Index test	New trauma team activation (TTA)
Reference standard	Later clinical confirmation of Major Trauma: ISS >15

van Laarhoven 2014

**Study_ID9**	**van Laarhoven 2014**
Study type	Retrospective analysis of prospectively collected data of all high-energy trauma patients
Number of studies/participants	1.607 adult patients
Countries and Settings	Region Central Netherlands
Funding	Not reported
Duration of study	from 2008 to 2011
Age, gender, ethnicity	(M: F) Not reported ; Age: Not reported ; Ethnicity: Not reported
Patient characteristics	Highest emergency and were over 17 years of age.
Index test	Dutch field triage protocol (ASC-COT)
Reference standard	Later clinical confirmation of Major Trauma: ISS >15

Bouzat 2015

**Study _ID 10**	**Bouzat 2015**
Study type	Retrospective data (register TRENAU) but prospectively collected
Number of studies/participants	3.428 (Of these, 2.552 patients were referred to Level-I or Level-II trauma centers, and 876 patients were admitted to Level-III centers)
Countries and Settings	The TRENAU federates 22 hospitals within a regional area (Figure 1), of which 13 are designated as Level I, II or III trauma centers depending on their technical facilities, France
Funding	Not reported
Duration of study	Three-year period (2009 to 2011)
Age, gender, ethnicity	(M:F) 3:1; Age (mean± SD): 37 ± 19; Ethnicity: Not reported
Patient characteristics	Severe trauma was suspected in the pre-hospital setting using the French Vittel triage criteria
Index test	ACSCOT, TRENAU
Reference standard	Later clinical confirmation of Major Trauma: ISS >15

Bouzat 2016

**Study**	**Bouzat 2016**
Study type	Retrospective data (register TRENAU) but prospectively collected
Number of studies/participants	3.260 patients
Countries and Settings	The TRENAU federates 22 hospitals within a regional area (Figure 1), of which 13 are designated as Level I, II or III trauma centers depending on their technical facilities, France
Funding	Not reported
Duration of study	three-year period (2009-2011)
Age, gender, ethnicity	(M: F) 3:1; Age (mean± SD): 37 ± 19; Ethnicity: Not reported
Patient characteristics	Patients with severe trauma suspected in the pre-hospital setting using the French Vittel triage criteria.
Index test	MGAP, T-RTS and TRISS (not applicable in pre-hospital setting)
Reference standard	Later clinical confirmation of Major Trauma: intra-hospital mortality (time point not reported)

Cassignol 2019

**Study_ID12**	**Cassignol 2019 a**
Study type	Monocentric retrospective study
Number of studies/participants	1.001
Countries and Settings	Level 1 trauma center in southern France
Funding	Not reported
Duration of study	Over a 4-year period (2013–2016)
Age, gender, ethnicity	(M: F) 4:1; Age (media ± SD): 43± 19; Ethnicity: Not reported
Patient characteristics	Patients are included if severe trauma was suspected in the prehospital setting
Index test	T-RTS= Triage Revised Trauma Score;<br>Vittel triage criteria;<br>MGAP= Mechanism, Glasgow Coma Scale, Age, systolic arterial Pressure;<br>NTS = New Trauma Score.
Reference standard	Later clinical confirmation of Major Trauma: ISS >15: intra-hospital mortality at 30 days

Sewalt 2019

**Study**	**Sewalt 2019**
Study type	Validation cohort
Number of studies/participants	Adult patients n=154.476, TARN registry (in-hospital mortality (11.882 patients) and major trauma (52 818))
Countries and Settings	The TARN database is the national trauma registry of England, Wales, Northern Ireland and the Republic of Ireland, with some members in continental Europe.
Funding	Not reported
Duration of study	Between 2013 and 2016
Age, gender, ethnicity	(M: F) 2:1; Age, median (range): 61 (39–81); Ethnicity: Not reported
Patient characteristics	
Index test	PHI, Prehospital Index; T-RTS, Triage Revised Trauma Score; PSS, Physiologic Severity Score; MGAP, Mechanism, Glasgow Coma Scale, Age and Arterial Pressure; mREMS, modified Rapid Emergency Medicine Score; KTS, Kampala Trauma Score.
Reference standard	Later clinical confirmation of Major Trauma: ISS >15<br>Later clinical confirmation of Major Trauma: intra-hospital mortality (time point not reported)

Llompart-Pou 2016

**Study**	**Llompart-Pou 2016**
Study type	Retrospective data (register RETRAUCI) but prospectively collected
Number of studies/participants	1.361
Countries and Settings	34 participating ICUs collecting data from trauma patients on a web-based system, Spain
Funding	Fundación Mutua Madrileña, GT Trauma y Neurointensivismo SEMICYUC
Duration of study	Three-year period (November 2012 to July 2015)
Age, gender, ethnicity	(M: F) 4:1; Age (median, range): 45 (30–61); Ethnicity: Not reported
Patient characteristics	All patients admitted for traumatic disease in the participating ICUs
Index test	T-RTS, MGAP, GAP, TRISS (not applicable in pre-hospital setting)
Reference standard	Later clinical confirmation of Major Trauma: Survival

Cassignol 2019 b

**Study**	**Cassignol 2019 b**
Study type	Retrospective data but prospectively collected
Number of studies/participants	1151
Countries and Settings	Sainte Anne Military Hospital of Toulon (South East of France), Level I Trauma Center
Funding	Intercommunal hospital center of Toulon and La Seyne
Duration of study	Four-year period (January 2013 to September 2016)
Age, gender, ethnicity	(M: F) 4:1; Age (mean, SD): 43 years (± 19); Ethnicity: Not reported
Patient characteristics	All patients admitted for traumatic disease
Index test	Vittel Triage Criteria
Reference standard	Later clinical confirmation of Major Trauma: ISS >15

Supplement C. Accuracy data of pre-hospital tools

LEGEND in Adults tools

1. American College of Surgeons' Committee on Trauma (ACS-COT)
2. Dutch Field Triage Protocol (ACS-COT)
3. Glasgow Coma Scale, Age, systolic arterial Pressure (GAP)
4. Kampala Truama Score (KTS)
5. Mechanism, Glasgow Coma Scale, Age, systolic arterial Pressure (MGAP)
6. modified Rapid Emergency Medicine Score (mREMS)
7. New Trauma team activation criteria (New TTA)
8. New Trauma Score (NTS)
9. Pre-Hospital Index (PHI)
10. Physiologic Severity Score (PSS)
11. Trauma system of the Northern French Alps(TRENAU)
12. Triage Revised Triage Score (T-RTS)
13. Vittel Triage Criteria

LEGEND in Children tools

1. East Midlands
2. London
3. North West
4. Northern
5. Paediatric Trauma Score (PTS)
6. Paediatric Triage Tape (PTT)
7. South West London
8. Wessex
9. CareFlight
10. JumpSTART/START
11. Triage Sort

Figure 2.A Forest plot - Accuracy trauma tools test in adults (ISS)

ASC-COT (ISS >15 as reference) in adults

Study	TP	FP	FN	TN	Sensitivity (95% CI)	Specificity (95% CI)
Bouzat 2015	976	1047	209	340	0.82 [0.80, 0.84]	0.25 [0.22, 0.27]
Dinh 2012	180	587	105	1792	0.63 [0.57, 0.69]	0.75 [0.74, 0.77]
Do 2014	139	45	43	1469	0.76 [0.70, 0.82]	0.97 [0.96, 0.98]
Ocak 2009	127	34	24	117	0.84 [0.77, 0.90]	0.77 [0.70, 0.84]
van Laarhoven 2014	197	547	24	839	0.89 [0.84, 0.93]	0.61 [0.58, 0.63]
Voskens 2018	342	94	1382	3132	0.20 [0.18, 0.22]	0.97 [0.96, 0.98]

ASC-COT (ISS>15 as reference) in elderly

Study	TP	FP	FN	TN	Sensitivity (95% CI)	Specificity (95% CI)
Voskens 2018	81	201	51	752	0.61 [0.52, 0.70]	0.79 [0.76, 0.81]

New TTA (ISS>15 as reference) in adults

Study	TP	FP	FN	TN	Sensitivity (95% CI)	Specificity (95% CI)
Vinjevoll 2018	0	758	0	113	Not estimable	0.13 [0.11, 0.15]

TRENAU (ISS>15 as reference) in adults

Study	TP	FP	FN	TN	Sensitivity (95% CI)	Specificity (95% CI)
Bouzat 2015	1090	818	95	569	0.92 [0.90, 0.93]	0.41 [0.38, 0.44]

Vittel Triage Criteria (ISS>15 as reference) in adults

Study	TP	FP	FN	TN	Sensitivity (95% CI)	Specificity (95% CI)
Follin 2016	0	476	0	267	Not estimable	0.36 [0.32, 0.40]

T-RTS ≤12 (ISS>15 as reference) in adults

Study	TP	FP	FN	TN	Sensitivity (95% CI)	Specificity (95% CI)
Sewalt 2019	17536	8234	35282	93424	0.33 [0.33, 0.34]	0.92 [0.92, 0.92]

MGAP ≤28 (ISS >15 as reference) in adults

Study	TP	FP	FN	TN	Sensitivity (95% CI)	Specificity (95% CI)
Sewalt 2019	36444	52049	16374	49609	0.69 [0.69, 0.69]	0.49 [0.48, 0.49]

PHI ≥ 1 (ISS>15 as reference) in adults

Study	TP	FP	FN	TN	Sensitivity (95% CI)	Specificity (95% CI)
Sewalt 2019	32272	24093	20546	77565	0.61 [0.61, 0.62]	0.76 [0.76, 0.77]

PSS≤11 (ISS>15 as reference) in adults

Study	TP	FP	FN	TN	Sensitivity (95% CI)	Specificity (95% CI)
Sewalt 2019	31427	21653	21391	80005	0.60 [0.59, 0.60]	0.79 [0.78, 0.79]

mREMS >3 (ISS>15 as reference) in adults

Study	TP	FP	FN	TN	Sensitivity (95% CI)	Specificity (95% CI)
Sewalt 2019	40617	73600	12201	28058	0.77 [0.77, 0.77]	0.28 [0.27, 0.28]

KTS ≤15 (ISS>15 as reference) in adults

Study	TP	FP	FN	TN	Sensitivity (95% CI)	Specificity (95% CI)
Sewalt 2019	50917	84173	1901	17485	0.96 [0.96, 0.97]	0.17 [0.17, 0.17]

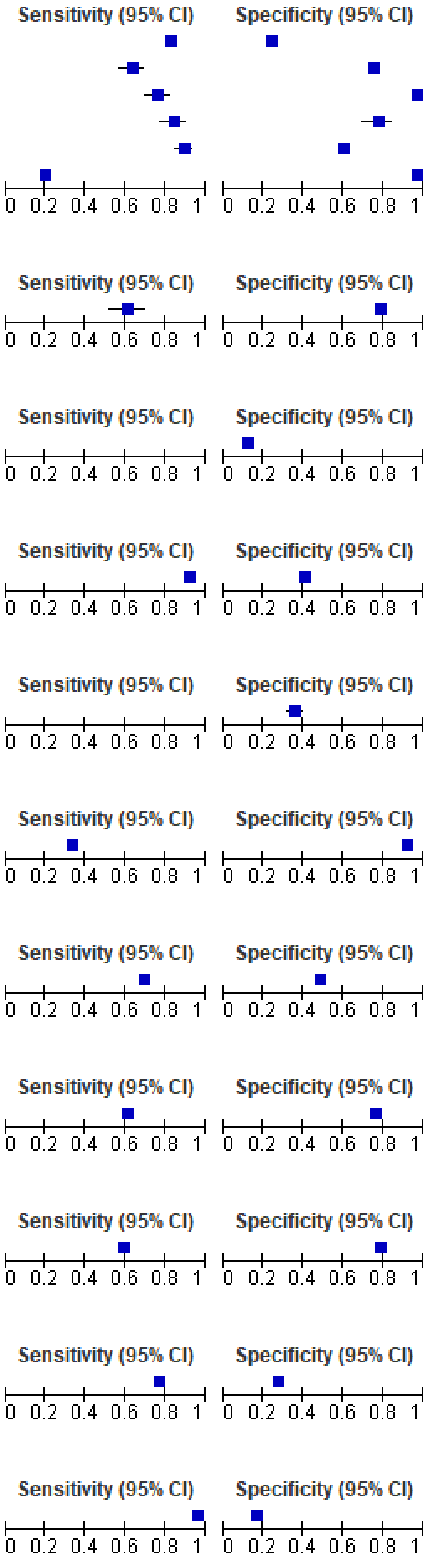

Figure 2.B Forest plot -Accuracy trauma tools test in adults (mortality, survival and ICU length of stay)

MGAP <23 (intra-hospital mortality as reference) in adults

Study	TP	FP	FN	TN	Sensitivity (95% CI)	Specificity (95% CI)
Bouzat 2016	164	553	22	2521	0.88 [0.83, 0.92]	0.82 [0.81, 0.83]
Cassignol 2019	69	222	7	703	0.91 [0.82, 0.96]	0.76 [0.73, 0.79]

NTS <18 (intra-hospital mortality as reference) in adults

Study	TP	FP	FN	TN	Sensitivity (95% CI)	Specificity (95% CI)
Cassignol 2019	62	130	14	796	0.82 [0.71, 0.90]	0.86 [0.84, 0.88]

T-RTS <12 (intra-hospital mortality as reference) in adults

Study	TP	FP	FN	TN	Sensitivity (95% CI)	Specificity (95% CI)
Bouzat 2016	147	369	39	2705	0.79 [0.72, 0.85]	0.88 [0.87, 0.89]
Cassignol 2019	69	601	7	324	0.91 [0.82, 0.96]	0.35 [0.32, 0.38]

Vittel Triage Criteria ≥ 1 (intra-hospital mortality as reference) in adults

Study	TP	FP	FN	TN	Sensitivity (95% CI)	Specificity (95% CI)
Cassignol 2019	76	907	0	19	1.00 [0.95, 1.00]	0.02 [0.01, 0.03]

MGAP<14.5 (intra-hospital survival as reference) in adults

Study	TP	FP	FN	TN	Sensitivity (95% CI)	Specificity (95% CI)
Llompart-Pou 2016	1098	152	22	89	0.98 [0.97, 0.99]	0.37 [0.31, 0.43]

GAP <11.5 (intrahospital survival as reference) in adults

Study	TP	FP	FN	TN	Sensitivity (95% CI)	Specificity (95% CI)
Llompart-Pou 2016	1064	130	56	111	0.95 [0.94, 0.96]	0.46 [0.40, 0.53]

T-RTS <7.5 (intra-hospital survival as reference) in adults

Study	TP	FP	FN	TN	Sensitivity (95% CI)	Specificity (95% CI)
Llompart-Pou 2016	1086	164	34	77	0.97 [0.96, 0.98]	0.32 [0.26, 0.38]

MGAP ≤ 22 (ICU admission as reference) in adults

Study	TP	FP	FN	TN	Sensitivity (95% CI)	Specificity (95% CI)
Follin 2016	124	62	352	623	0.26 [0.22, 0.30]	0.91 [0.89, 0.93]

MGAP ≤ 17 (ICU admission as reference) in adults

Study	TP	FP	FN	TN	Sensitivity (95% CI)	Specificity (95% CI)
Follin 2016	43	14	432	671	0.09 [0.07, 0.12]	0.98 [0.97, 0.99]

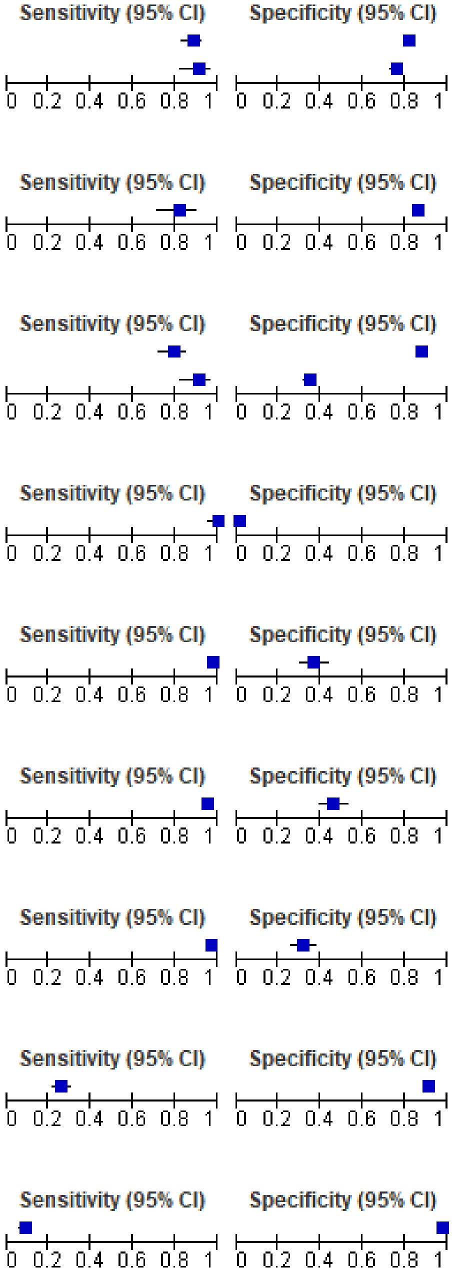

Figure 3.A SROC plot - Accuracy trauma tools tests in adults (ISS> 15)

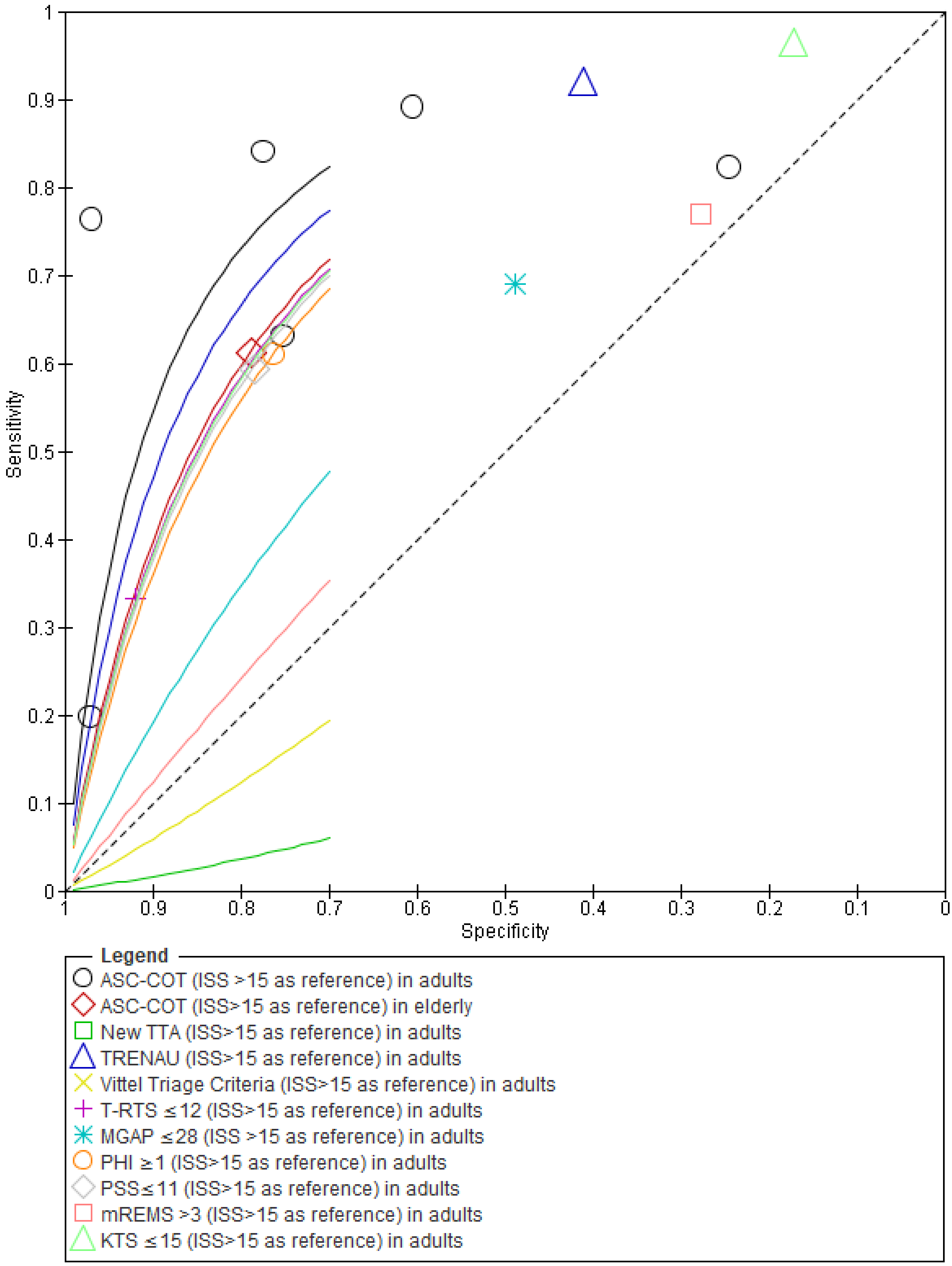

Figure 3.B SROC plot - Accuracy trauma tools tests in adults (mortality, survival and ICU length of stay)

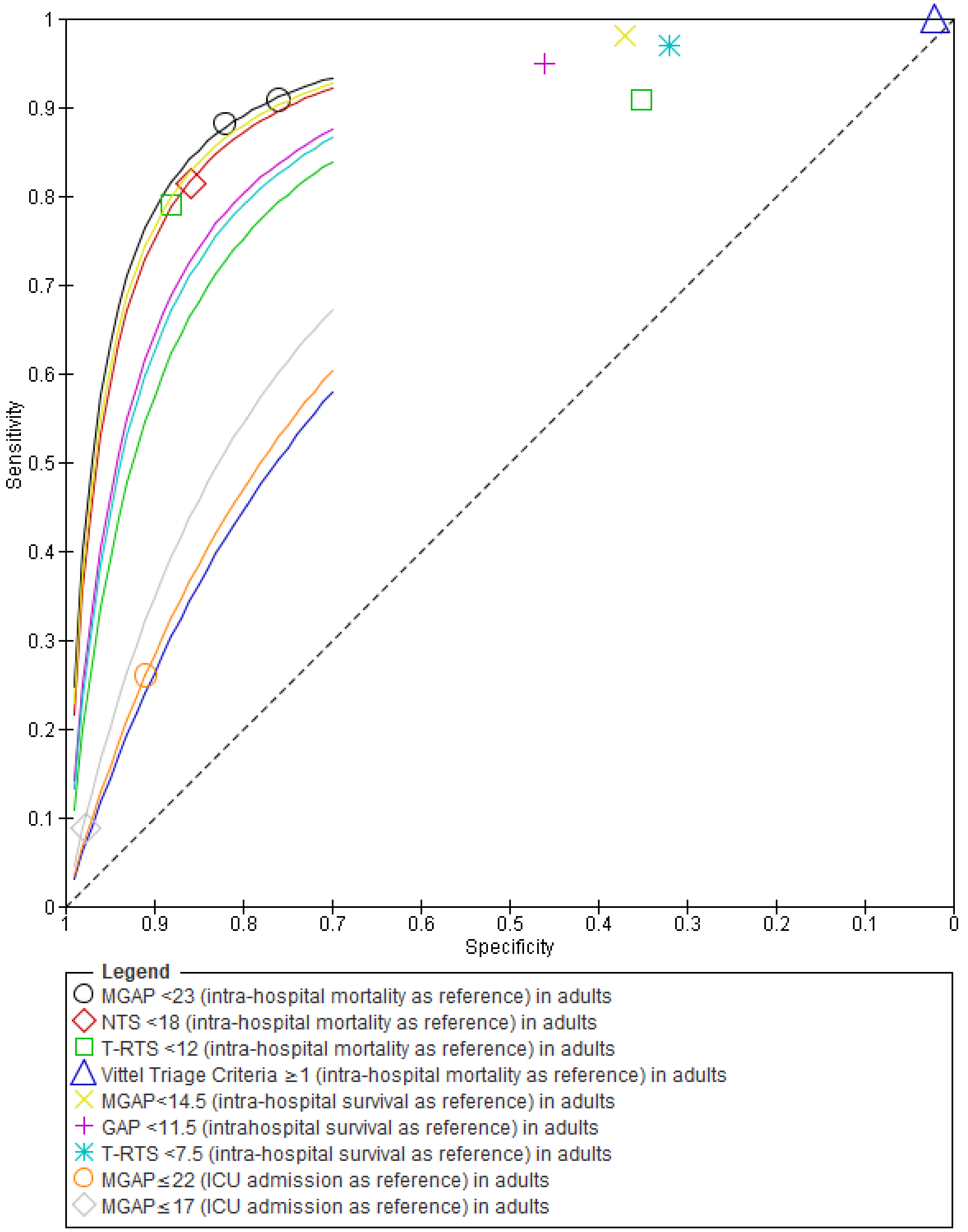

Figure 4. Metanalysis- index test ACS-COT in adults (reference test ISS>15)

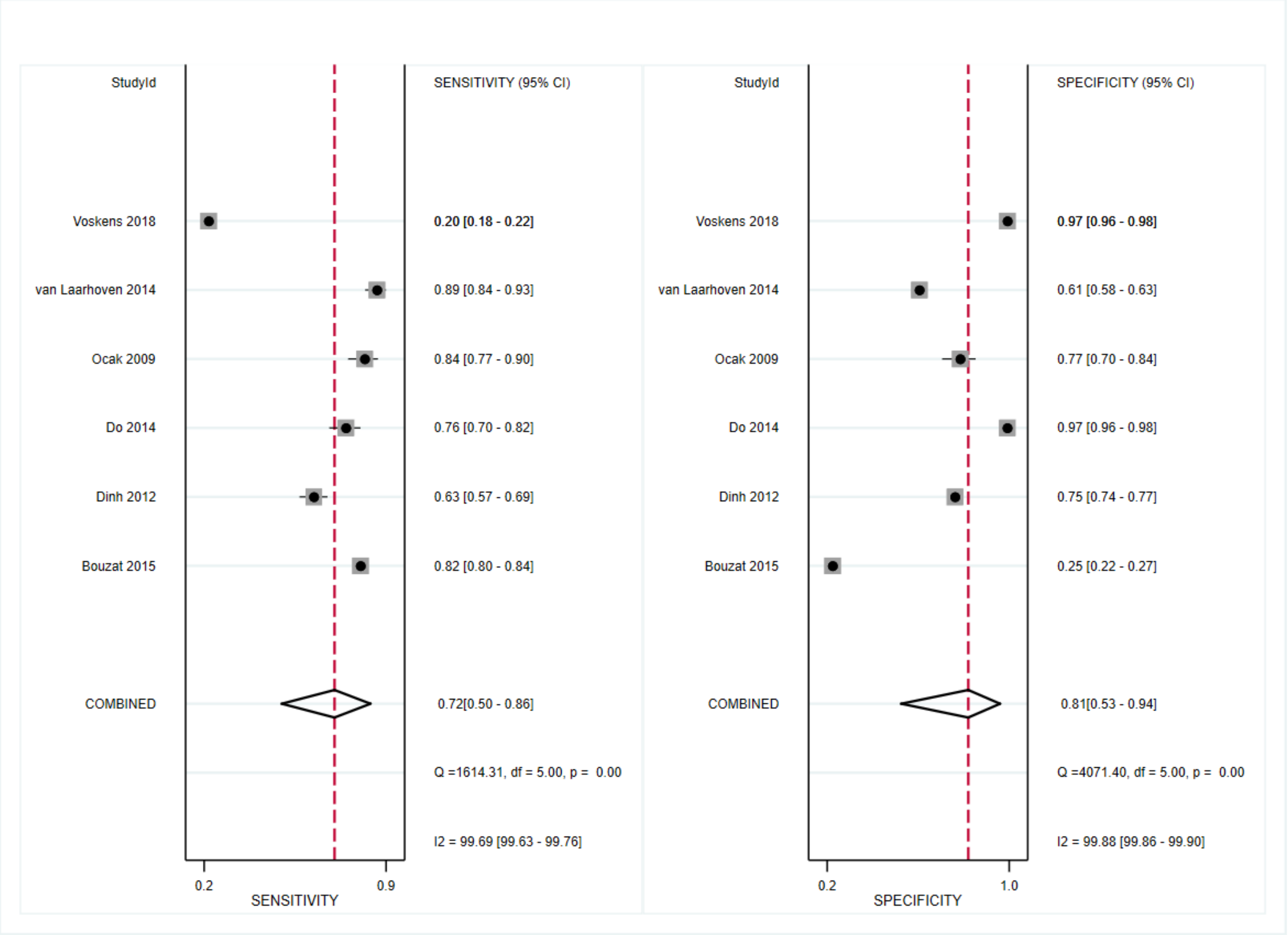

Note: Due to highest heterogeneity (I^2=99%), the combined estimate of sensitivity and specificity was not included in the GRADE summary of findings table.

Figure 5. Forest plot - Accuracy trauma tools test in children

CareFlight (ISS>15 as reference) in children

Study	TP	FP	FN	TN	Sensitivity (95% CI)	Specificity (95% CI)
Price 2016	4413	2494	2429	21956	0.64 [0.63, 0.66]	0.90 [0.89, 0.90]

East Midlands (ISS > 15 as reference) in children

Study	TP	FP	FN	TN	Sensitivity (95% CI)	Specificity (95% CI)
Cheung 2013	223	391	7	80	0.97 [0.94, 0.99]	0.17 [0.14, 0.21]

JumpSTART/START (ISS > 15 as reference) in children

Study	TP	FP	FN	TN	Sensitivity (95% CI)	Specificity (95% CI)
Price 2016	4078	5795	2764	18655	0.60 [0.58, 0.61]	0.76 [0.76, 0.77]

London (ISS > 15 as reference) in children

Study	TP	FP	FN	TN	Sensitivity (95% CI)	Specificity (95% CI)
Cheung 2013	221	339	9	132	0.96 [0.93, 0.98]	0.28 [0.24, 0.32]

North West (ISS > 15 as reference) in children

Study	TP	FP	FN	TN	Sensitivity (95% CI)	Specificity (95% CI)
Cheung 2013	214	377	16	94	0.93 [0.89, 0.96]	0.20 [0.16, 0.24]

Northen (ISS > 15 as reference) in children

Study	TP	FP	FN	TN	Sensitivity (95% CI)	Specificity (95% CI)
Cheung 2013	209	363	21	108	0.91 [0.86, 0.94]	0.23 [0.19, 0.27]

Paediatric Trauma Score (ISS>15 as reference) in children

Study	TP	FP	FN	TN	Sensitivity (95% CI)	Specificity (95% CI)
Cheung 2013	90	33	140	438	0.39 [0.33, 0.46]	0.93 [0.90, 0.95]

Paediatric Triage Tape (ISS>15 as reference) in children

Study	TP	FP	FN	TN	Sensitivity (95% CI)	Specificity (95% CI)
Cheung 2013	34	30	60	159	0.36 [0.27, 0.47]	0.84 [0.78, 0.89]
Price 2016	2490	8191	4352	16259	0.36 [0.35, 0.38]	0.66 [0.66, 0.67]

South West London (ISS>15 as reference) in children

Study	TP	FP	FN	TN	Sensitivity (95% CI)	Specificity (95% CI)
Cheung 2013	202	278	28	193	0.88 [0.83, 0.92]	0.41 [0.36, 0.46]

Triage Sort (ISS>15 as reference) in children

Study	TP	FP	FN	TN	Sensitivity (95% CI)	Specificity (95% CI)
Price 2016	4830	5355	2012	19095	0.71 [0.69, 0.72]	0.78 [0.78, 0.79]

Wessex (ISS>15 as reference) in children

Study	TP	FP	FN	TN	Sensitivity (95% CI)	Specificity (95% CI)
Cheung 2013	177	250	53	221	0.77 [0.71, 0.82]	0.47 [0.42, 0.52]

CareFlight (mortality as reference) in children

Study	TP	FP	FN	TN	Sensitivity (95% CI)	Specificity (95% CI)
Price 2016	981	5932	48	24331	0.95 [0.94, 0.97]	0.80 [0.80, 0.81]

JumpSTART/START (mortality as reference) in children

Study	TP	FP	FN	TN	Sensitivity (95% CI)	Specificity (95% CI)
Price 2016	945	8928	84	21335	0.92 [0.90, 0.93]	0.70 [0.70, 0.71]

Paediatric Triage tape (mortality as reference) in children

Study	TP	FP	FN	TN	Sensitivity (95% CI)	Specificity (95% CI)
Price 2016	389	10289	640	19974	0.38 [0.35, 0.41]	0.66 [0.65, 0.67]

Triage Sort (mortality as reference) in children

Study	TP	FP	FN	TN	Sensitivity (95% CI)	Specificity (95% CI)
Price 2016	990	9200	39	21063	0.96 [0.95, 0.97]	0.70 [0.69, 0.70]

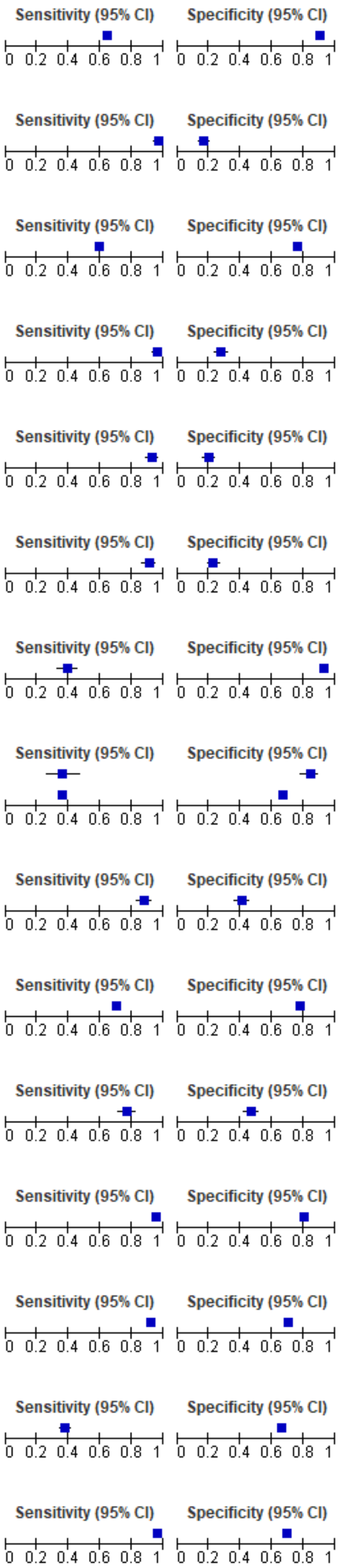

Figure 6. SROC plot- Accuracy trauma tools test in children

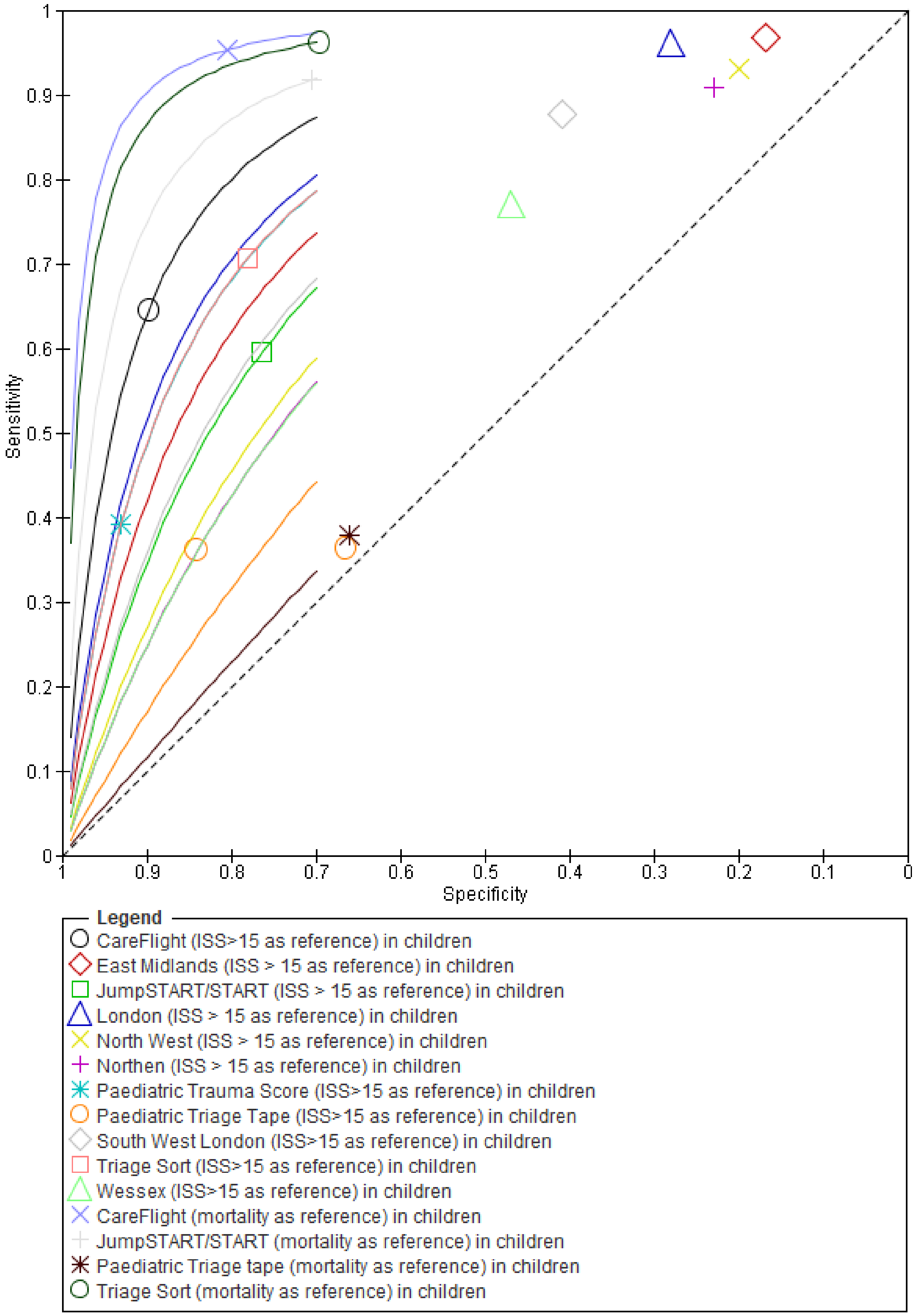

Table 1. Under triage and over triage of triage trauma tools in children

Study ID	INDEX tool	Total	ISS > 15	Undertriage (%)	Overtriage (%)
**Index test *vs reference (*ISS >15)**					
Price 2016	CareFlight	3.1292	6842	35,5	10,2
Cheung 2013	East Midlands	701	230	3	83
Price 2016	JumpSTART/START	31.292	6.842	40,4	23,7
Cheung 2013	London	701	230	4	72
Cheung 2013	North West	701	230	7	80
Cheung 2013	Northern	701	230	9	77
Cheung 2013	Paediatric Trauma Score	701	230	61	7
Cheung 2013	Paediatric Triage Tape	283	94	63	16
Price 2016	Paediatric Triage Tape	31.292	6.842	63,6	33,5
Cheung 2013	South West Londo	701	230	12	59
Price 2016	Triage Sort	31.292	6.842	29,4	21,9
Cheung 2013	Wessex	701	230	23	53
**Index test *vs reference (*mortality)**					
**Study ID**	**INDEX tool**	**Total**	**Survival (alive)**	**Undertriage (%)**	**Overtriage (%)**
Price 2016	CareFlight	31.292	30.263	4,7	19,6
Price 2016	JumpSTART/START	31.292	30.263	8,2	29,5
Price 2016	Paediatric Triage tape	31.292	30.263	62,2	34
Price 2016	Triage Sort	31.292	30.263	3,8	30,4

Table 2. Predictive and negative values in adults with major trauma (ISS >15)

Index test *vs reference* (ISS >15)					
**Study ID**	**INDEX tool**	**Total**	**N ISS > 15**	**Positive predictive value (%)**	**Negative predictive value (%)**
Dinh 2012	ASC-COT	2664	285	23,5	94,5
Do 2014	ASC-COT	1696	182	75,5	97,2
Ocak 2009	ASC-COT	302	151	78,9	82,9
Voskens 2018	Dutch Field Triage Protocol (ACS-COT)	4950	436	78,4	69,3
Voskens 2018 (elderly > 65)	Dutch Field Triage Protocol (ACS-COT)	1085	132	28,7	93,7
Bouzat 2015	ASCOT	2572	1185	48	61
Bouzat 2015	TRENAU	2572	1185	58	85
Follin 2016	Vittel Triage Criteria	1160	417	na	na
van Laarhoven 2014	Dutch field triage protocol (ACS-COT)	1607	na	26,5	97,2
Vinjevoll 2018	New Trauma team activation criteria	998	127	na	na
Sewalt 2016	PHI ≤ 1 of 20	154476	52818	57	79
Sewalt 2016	T-RTS ≤ 11 of 12	154476	52818	68	73
Sewalt 2016	PSS ≤ 11 of 12	154476	52818	59	79
Sewalt 2016	MGAP ≤ 28 of 29	154476	52818	41	75
Sewalt 2016	mREMS > 3 of 26	154476	52818	35	7
Sewalt 2016	KTS ≤ 15 of 16	154476	52818	37	9
**Index test *vs reference* (hospital mortality)**					
**Study ID**	**INDEX tool**	**Total**	**Death**	**Positive predictive value (%)**	**Negative predictive value (%)**
Cassignol 2019 a	MGAP < 23	1001	76	24	99
Cassignol 2019 a	NTS (New Trauma Score) < 18	1001	76	33	98
Cassignol 2019 a	T-RTS < 12	1001	76	10	98
Cassignol 2019 a	Vittel Triage Criteria ≥ 1	1001	76	8	100
Bouzat 2016	MGAP < 23	3260	186	26	99
Bouzat 2016	T-RTS < 12	3260	186	19	98
**Index test *vs reference* (survival)**					
**Study ID**	**INDEX tool**	**Total**	**Survival**	**Positive predictive value (%)**	**Negative predictive value (%)**
Llompart-Pou 2016	MGAP < 14,5	1361	1120	88	81
Llompart-Pou 2016	GAP < 11,5	1361	1120	89	66
Llompart-Pou 2016	T-RTS	1361	1120	87	68
**Index test *vs reference* (ICU admission)**					
**Study ID**	**INDEX tool**	**Total**	**ICU admission**	**Positive predictive value (%)**	**Negative predictive value (%)**
Follin 2016	MGAP < 22	1160	475	66	64
Follin 2016	MGAP < 17	1160	475	75	60

Table 3. Predictive and negative values in children with major trauma (ISS >15)

Study ID	INDEX tool	N of cases	ISS >15	Positive Predictive Value (%)	Negativepredictive value (%)
**Index test *vs reference* (ISS > 15)**					
Price 2016	CareFlight	31.292	6.842	64,8	90
Cheung 2013	East Midlands	701	230	36	91
Price 2016	JumpSTART/START	31.292	6.842	41,3	87,1
Cheung 2013	London	701	230	39	93
Cheung 2013	North West	701	230	36	86
Cheung 2013	Northern	701	230	37	85
Cheung 2013	Paediatric Trauma Score	701	230	74	76
Cheung 2013	Paediatric Triage Tape	283	94	53	73
Price 2016	Paediatric Triage Tape	31.292	6.842	23,3	78,9
Cheung 2013	South West Londo	701	230	42	87
Price 2016	Triage Sort	31.292	6.842	47	90,4
Cheung 2013	Wessex	701	230	42	81
**Index test *vs. reference* (in-hospital mortality)**					
**Study ID**	**INDEX tool**	**N of cases**	**ISS >15**	**Positive Predictive Value (%)**	**Negative predictive value (%)**
Price 2016	CareFlight	31.292	1.029	14,2	99,8
Price 2016	JumpSTART/START	31.292	1.029	95,7	99,6
Price 2016	Paediatric Triage tape	31.292	1.029	36,4	96,9
Price 2016	Triage Sort	31.292	1.029	97,2	98,2

Supplement D. Net clinical benefit curves

The net clinical benefit curve was calculated for all pre-hospital triage tools at different thresholds. The net benefit curve represents the potential gain in using a prediction model for the triage of trauma patients compared to sending all patients to the trauma center. Net clinical benefit is defined as the proportion of true positives - (proportion of false positives × weight). Weight (1) is defined as the threshold ratio (maximum number of patients mistakenly classifying yourself as having major trauma (false positives) to correctly classify 1 patient with major trauma (true positives). For example, a threshold of 0.2 means that the trauma center will accept 4 patients mistakenly classified as having major trauma (true positives defined as ISS> 15). Therefore, for the threshold of 0.2 the weight will be 1: 4 (**table 1**).

Table 1. Match threshold, weight, true positive: false positive patient ratio

Threshold	*Weight*	*TP:FP*
0	*0.00*	*0*
0.1	*0.11*	*1:10*
0.2	*0.25*	*1:4*
0.3	*0.43*	*2:5*
0.4	*0.67*	*2:3*
0.5	*1.00*	*1:1*

Looking at the graphs below, the x-axis shows the threshold, defined as the ratio between the number of true positives and false positives. The number of false positives decreases as the threshold increases. The y-axis shows the benefit, defined as the difference between the proportion of true positives and the proportion of false positives corrected for weight (ie. Threshold ratio). **Formula 2** shows the complete calculation.

$$\left(\frac{p_t}{1 - p_t}\right)$$

(1) Formula for defining weight

$$\frac{\text{True Positive Count} - \text{False Positive Count} \times \left(\frac{p_t}{1-p_t}\right)}{\text{Total Sample Size}}$$

(2) Formula to detect the net clinical benefit

Vickers 2008, Am Stat. 62(4): 314–320

Figure 1. Net Benefit Curves of triage tools in adults with ISS >15 as reference standard.

a) ASC-COT

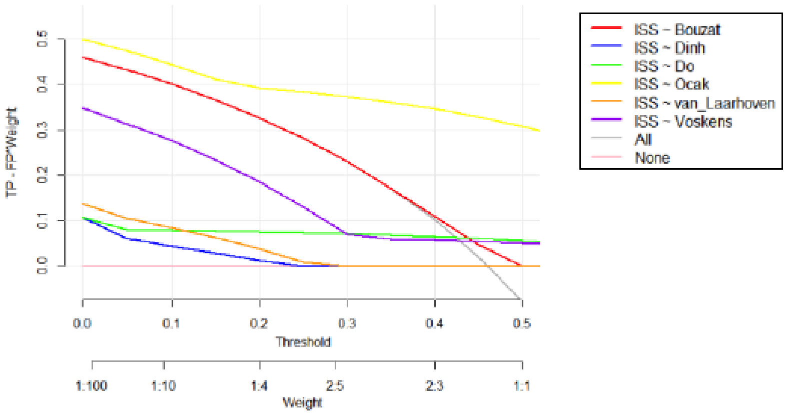

b) TRENAU (Sn 0.92; Sp 0.41)

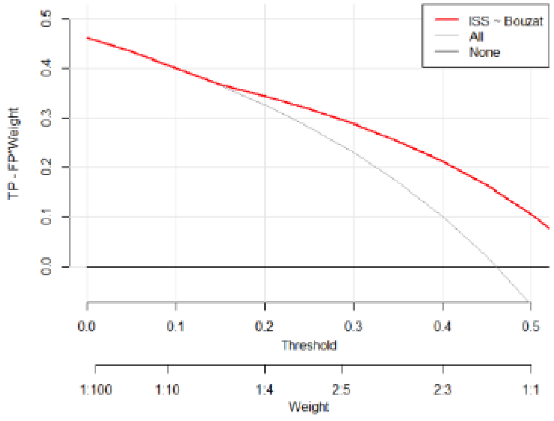

c) T-RTS (Sn 0.33, Sp 0.92)

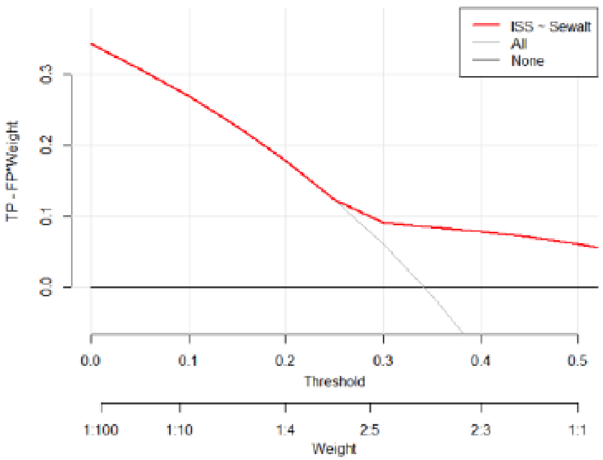

d) MGAP (Sn 0.69, Sp 0.49)

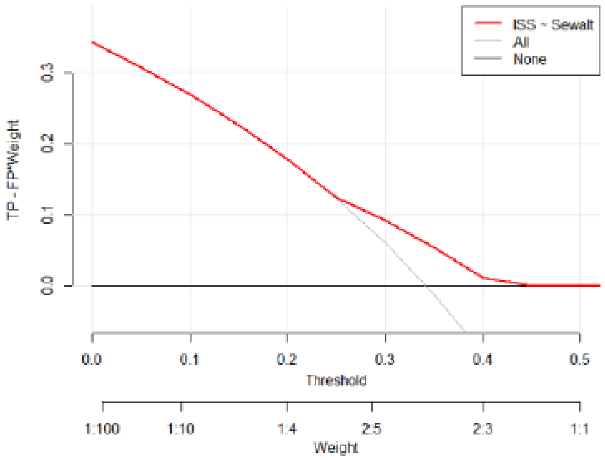

e) PHI (Sn 0.61, Sp 0.76)

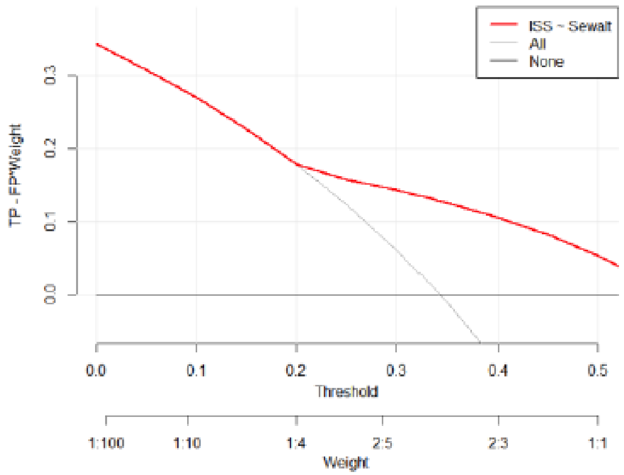

f) PSS (Sn 0.60, Sp 0.79)

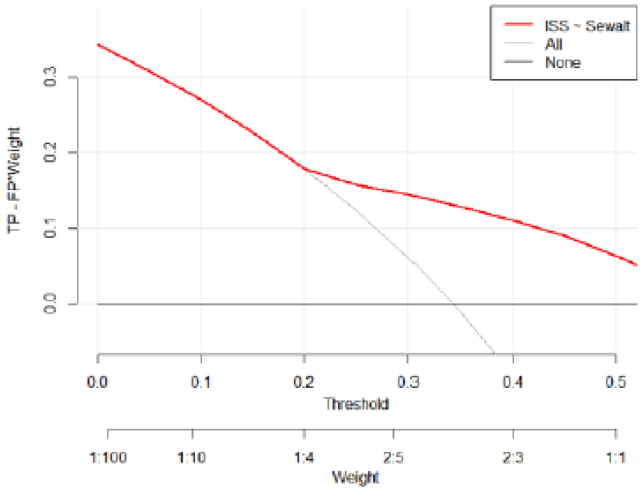

g) mREMS (Sn 0.77, Sp 0.28)

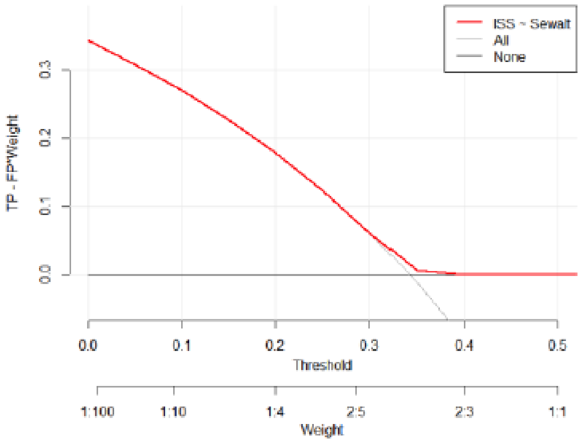

h) KTS (Sn 0.96, Sp 0.17)

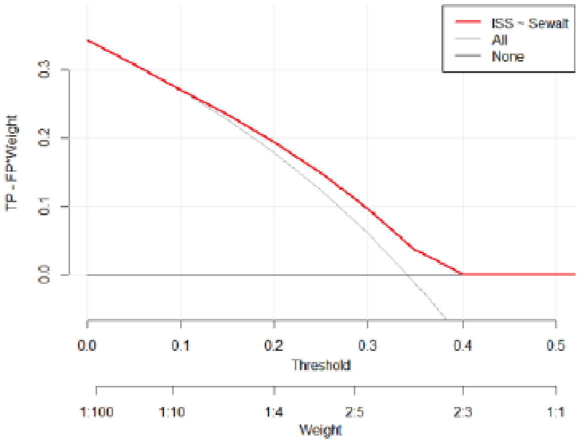

Figure2. Net Benefit curves of triage tools in adults with ISS >15 as reference standard.

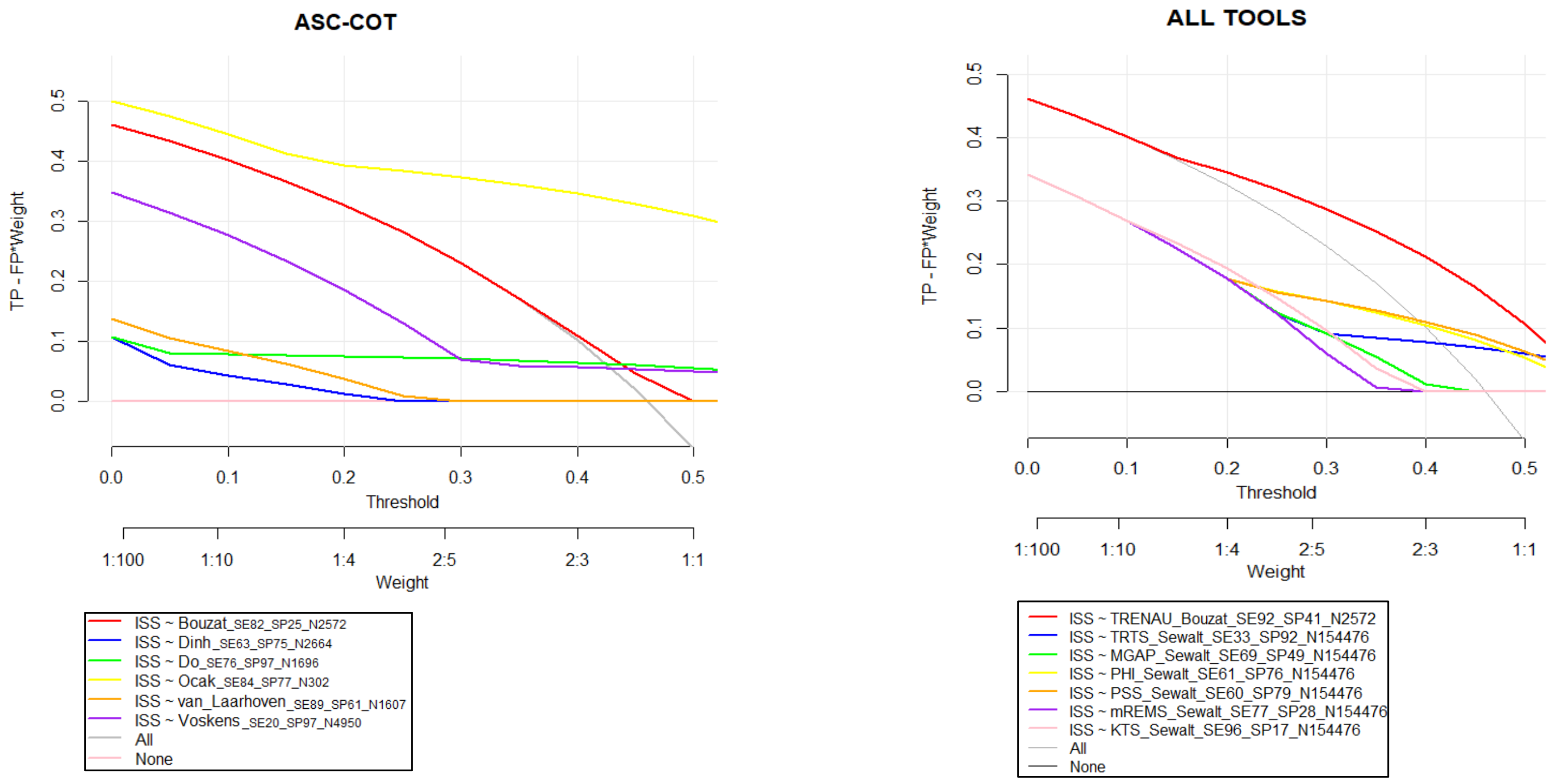

In adults, it was not possible to represent the net clinical benefit of 2 tools (New TTA and Vittel Triage Criteria) of the 10 pre-hospital triage tools found, compared to the severity index, since both did not report sensitivity and / or specificity data.

The ASC-COT tool shows highly variable curve trajectories for each type of study included (such as relative meta-analysis). This heterogeneity could be explained by the prevalence of mixed cases and by the subjectivity of some sections of the tool itself (e.g. high number of patients with impaired vital signs rather than with mechanism of injury).

The trajectory of the best curve seems to be that relating to the TRENAU tool (total number: 2572, values of Sn 0.92 and Sp 0.41, 1 study).

Figure 3. Net Benefit Curves of triage tools in adults with in-hospital mortality as reference standard.

MGAP<23 (Sn:0.88, Sp:0.82), (Sp:0.91, SP:0.76)

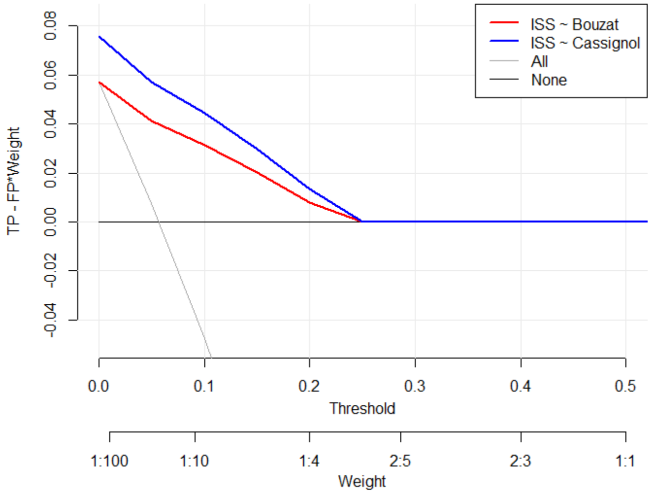

NTS <18 (Sn:0.82, Sp:0.86)

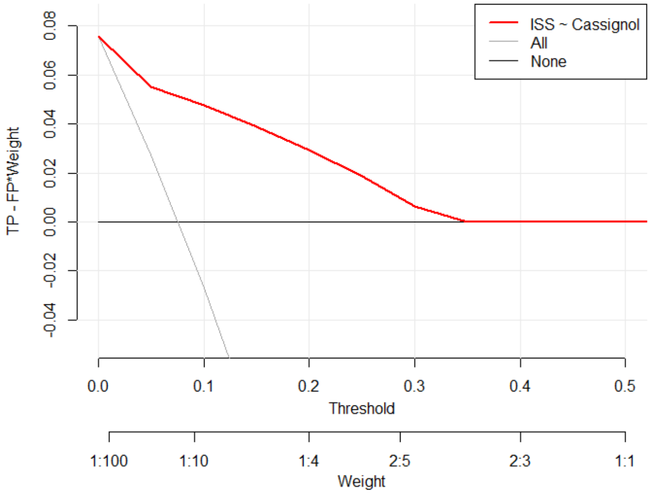

T-RTS<12 (Sn:0.79, Sp:0.88), (Sn:0.91, Sp:0.35)

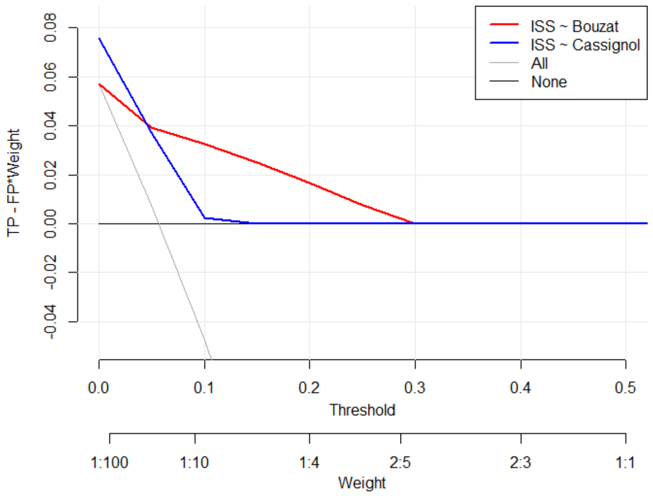

Vittel Triage Criteria (Sn:1.00, Sp:0.02)

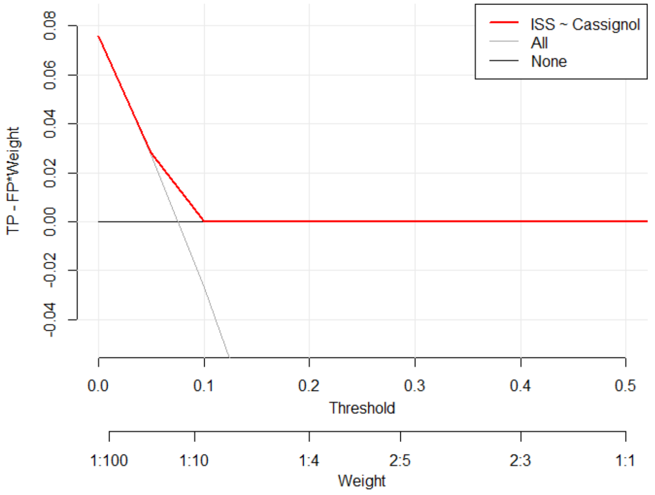

In adults, the pre-hospital triage tool with the best benefit trajectory is NTS <18 (Sn: 0.82, Sp: 0.86, 1 study), considering mortality as a reference standard.

Figure 4. Net Benefit Curves of triage tools in adults with in-hospital survival as reference standard.

MGAP<14.5 (Sn:0.98, Sp:0.37)

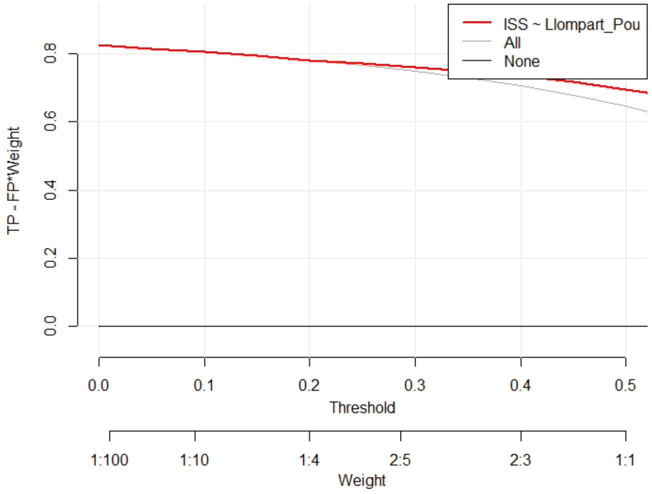

GAP<11.5 (Sn:0.95, Sp:0.46)

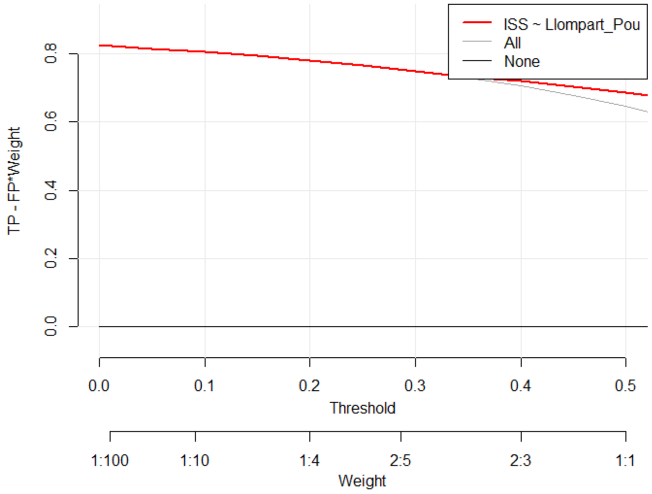

T-RTS<7.5 (Sn:0.97, Sp:0.32)

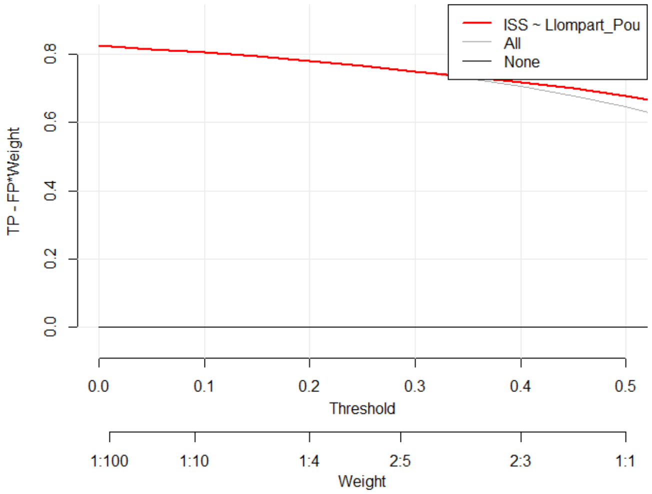

For the survival outcome, the three tools (MGAP <14.5, GAP <11.5, T-RTS <7.5) resulting from the Llompart Pou study do not show particular differences.

Figure 5. Net Benefit curves of triage tools in adults with ICU length of stay as reference standard.

MGAP<22 (Sn:0.26, Sp:0.91)

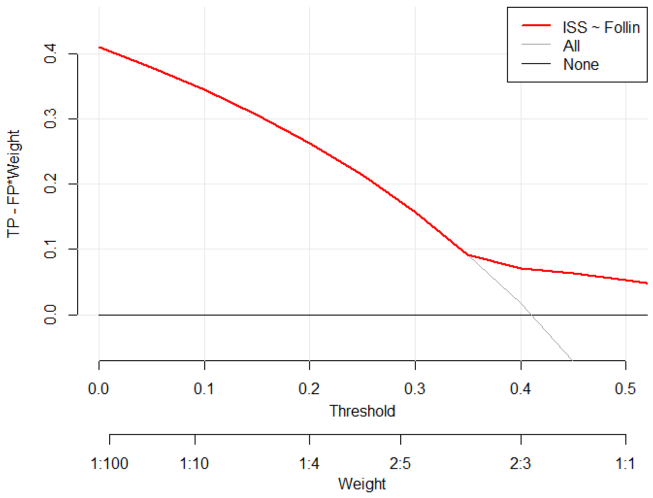

MGAP<17 (Sn:0.09, Sp:0.98)

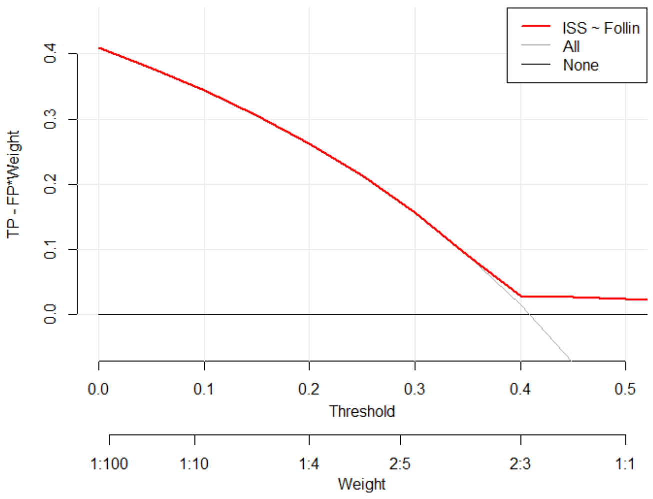

MGAP <22 appears to be better than MGAP <17 since, with the same high level of specificity, for the first tool the sensitivity is higher (0.26 vs 0.09).

Figure 6. Net Benefit Curves of pre-hospital triage tools in children with ISS> 15 as reference standard.

a)CareFlight (Sn:0.64, Sp:0.90)

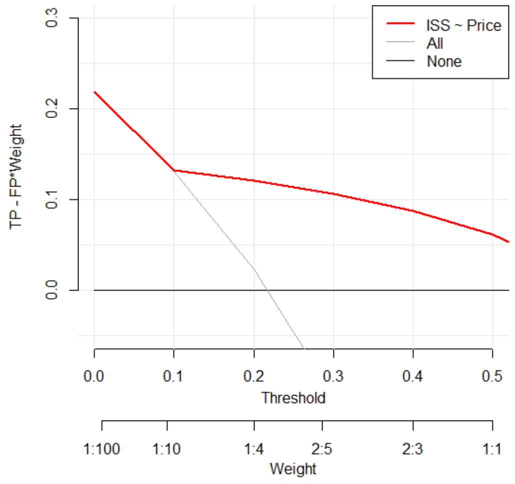

b)London (Sn:0.96, Sp:0.28)

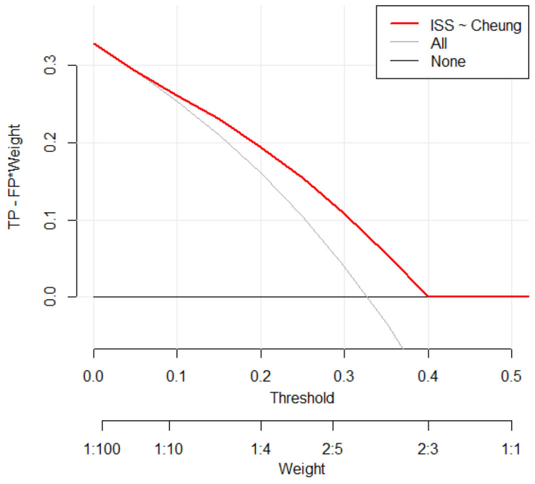

c)Paediatric Trauma Score (Sn:0.39, Sp:0.93)

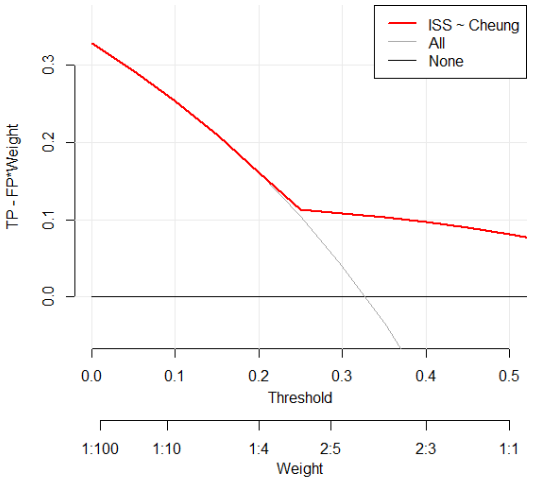

d)Triage Sort (Sn:0.71, Sp:0.78)

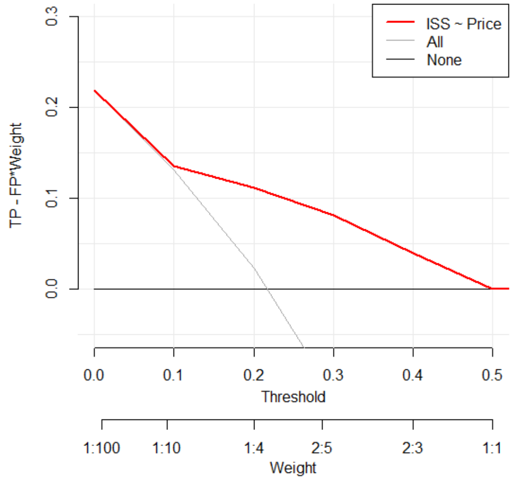

e)East Midlands (Sn:0.97, Sp:0.17)

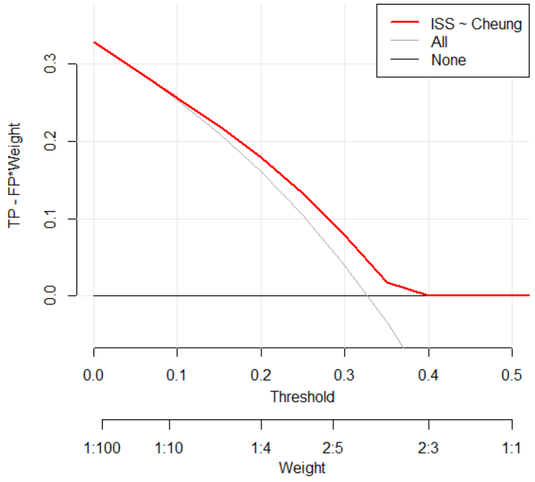

f)South West London (Sn 0.88, Sp: 0.41)

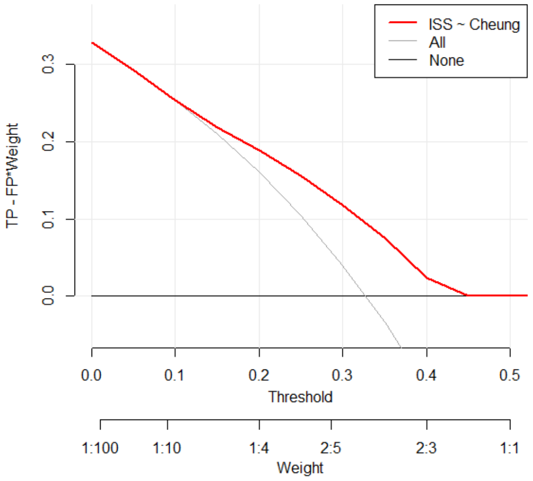

g)JumpSTART/START (Sn:0.60, Sp:0.76)

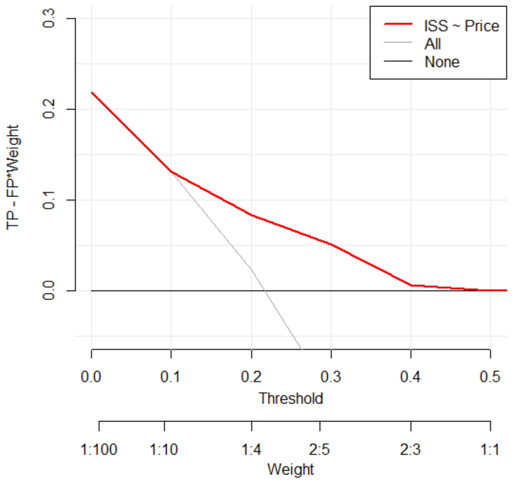

h)North West (Sn:0.93, Sp:0.20)

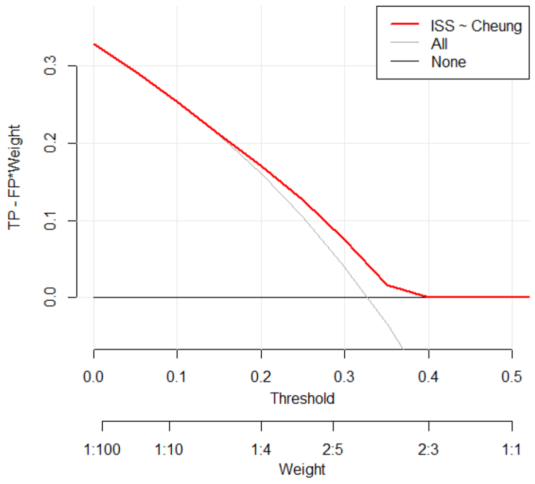

i)Wessex (Sn:0.77, Sp:0.47)

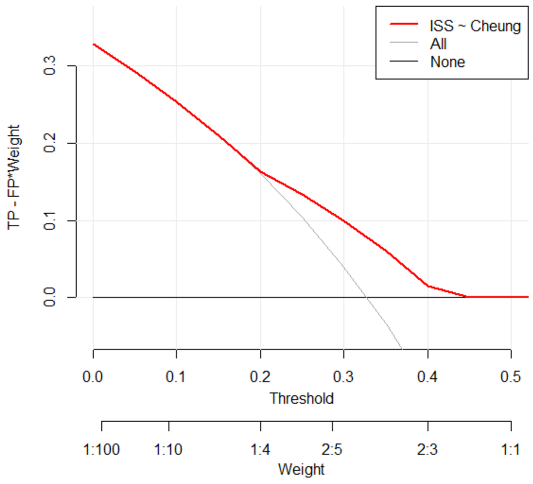

l) Northen (Sn:0.91, Sp:0.23)

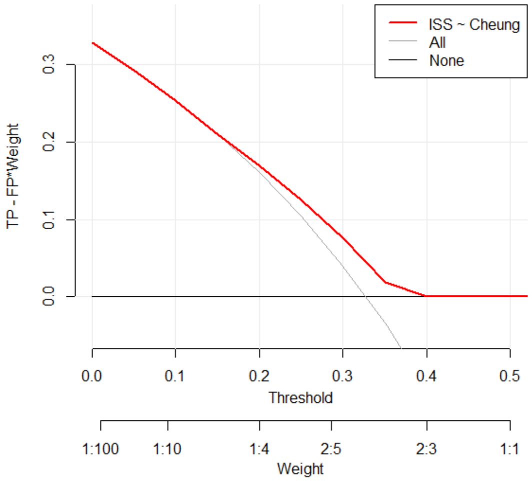

m) Paediatric Triage Tape (Sn:0.36, Sp:0.84) (Sn:0.36, Sp:0.66)

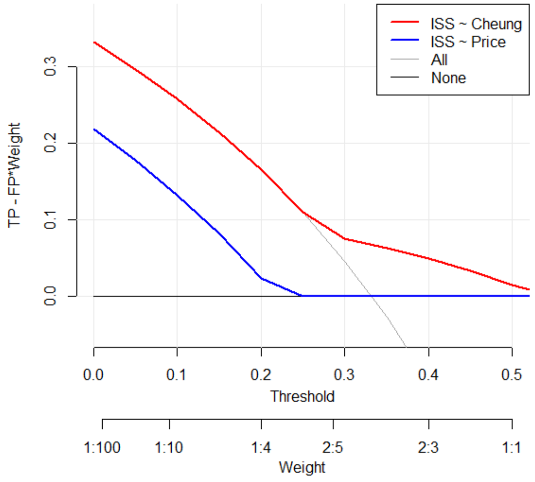

Figure 7. **Net Benefit Curves of pre-hospital triage tools in children with ISS >15 as reference standard.**

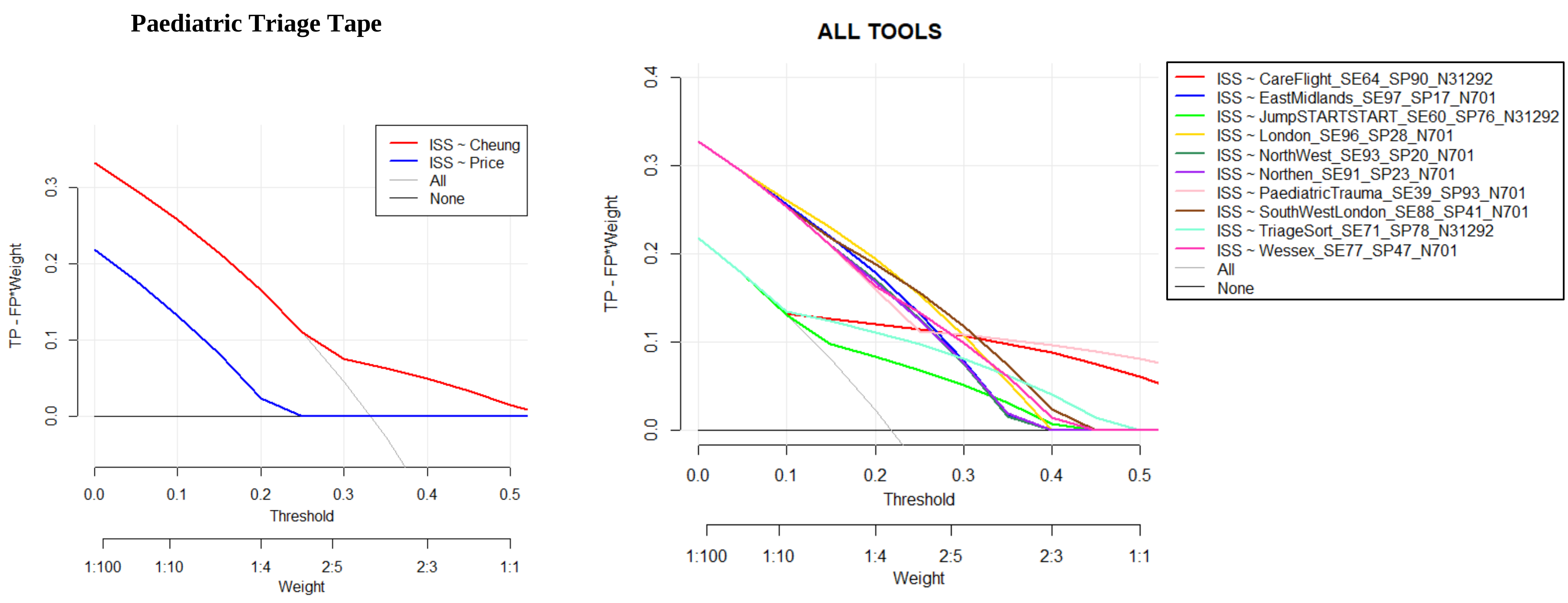

All the instruments considered were analyzed by only two studies whose sample size was different: respectively Price (n = 31292) and Cheung (n = 701). The CareFlight instrument seems to have the best net clinical benefit curve, as also demonstrated by the ROC curve which is the highest among all the tools (ROC Curve: Figure 6, Appendix C) while the Pediatric Triage Tape instrument appears to have the net worst clinical benefit and having the lowest ROC curve among all tools found (ROC Curve: Figure 6, Appendix C).

Figure 8. Net Benefit Curves of pre-hospital triage tools in children with intra-hospital mortality as reference standard.

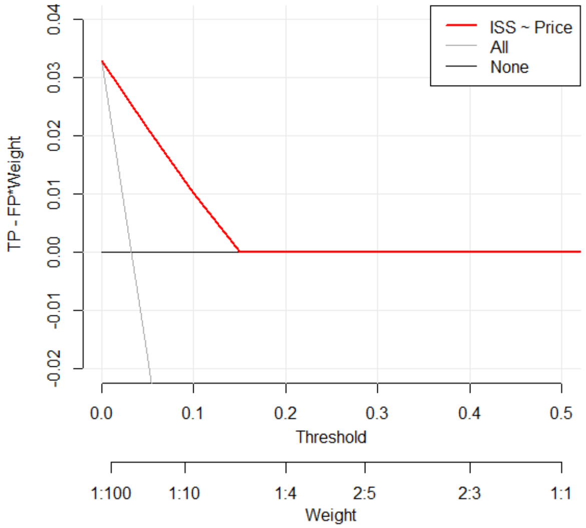

CareFlight (Sn:0.95, Sp:0.80)

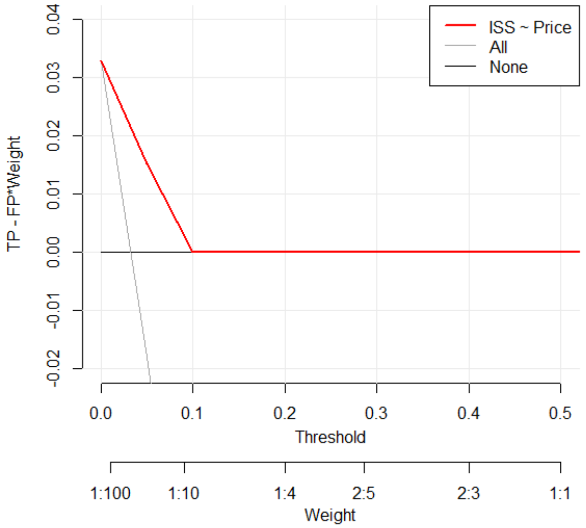

JumpSTART/START (Sn:0.92, Sp:0.70)

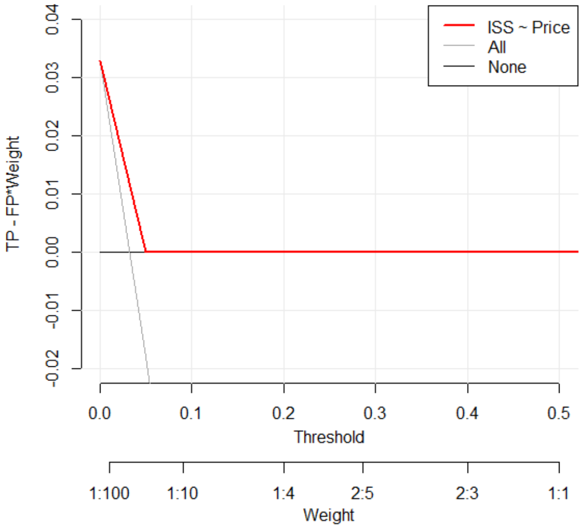

Paediatric Triage Tape (Sn:0.38, Sp:0.66)

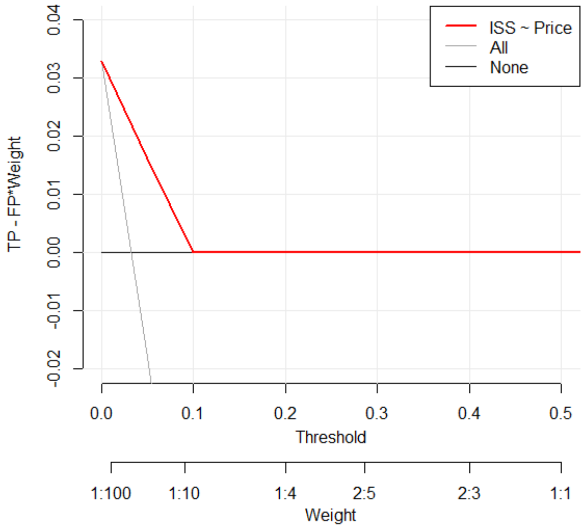

Triage Sort (Sn:0.96, Sp:0.70)

According to the curve ROC the CareFlight tool shown higher Sn e Sp, followed by the Triage Sort, JumpSTART/START e Paediatric Triage Tape tools.

Figure 9. Subgroup: Net Benefit Curves of pre-hospital triage tools in elderly with ISS >15 as reference standard

ASC-COT (Sn:0.61, Sp:0.79)

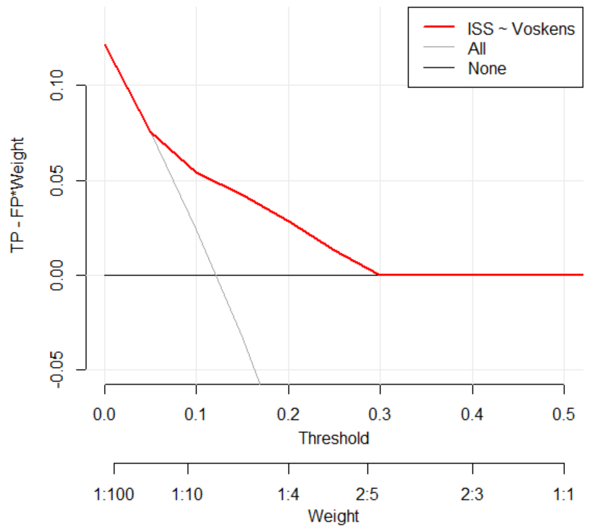

Supplement E. Quality assessment QUADAS 2

	Risk of bias				Applicability		
**Study – Author year**	**PATIENT SELECTION**	**INDEX TEST**	**REFERENCE STANDARD**	**FLOW AND TIMING**	**PATIENT SELECTION**	**INDEX TEST**	**REFERENCE STANDARD**
Cheun 2013	unclear	unclear	low	low	low	low	low
Do 2014	low	unclear	low	low	low	low	low
Dinh 2012	unclear	unclear	low	low	low	low	low
Ocak 2009	high	unclear	low	low	low	low	low
Follin 2016	low	unclear	low	low	low	low	low
Voskens 2018	high	unclear	low	low	low	low	low
Price 2016	low	unclear	low	low	low	low	low
Vinjevoll 2018	high	unclear	low	low	low	low	low
van Laarhoven 2014	low	unclear	low	low	low	low	low
Bouzat 2015	low	unclear	low	low	low	low	low
Bouzat 2016	low	low	low	low	low	low	low
Cassignol 2019a	low	low	low	low	low	low	low
Sewalt 2019	low	low	low	low	low	low	low
Cassignol 2019b	low	low	low	low	low	low	low
Llompart-Pou 2016	low	low	low	low	low	low	low

Supplement F. Summary of findings tables

Table 1: Summary of findings. Diagnostic accuracy of ACS-COT score in the prediction of major trauma in adults (standard of reference: ISS >15)

Sensitivity (median)	0.79 (95% CI: 0.73 to 0.83)
Specificity (median)	0.76 (95% CI: 0.72 to 0.81)

Prevalences	27%

Outcome	№ of studies (№ of patients)	Study design	Factors that may decrease certainty of evidence					Effect per 1.000 patients tested	Test accuracy CoE
			Risk of bias	Indirectness	Inconsistency	Imprecision	Publication bias	pre-test probability of 27%	
**True positives**<br>(patients with MAJOR TRAUMA)	6 studies<br>(N = 3.748)	cross-sectional (cohort type accuracy study)	Serious[a]	Not important	Serious[b]	Not important	None	213 (198 to 224)	⊕⊕◯◯<br>LOW
**False negatives**<br>(patients incorrectly classified as not having MAJOR TRAUMA)								57 (46 to 72)	
**True negatives**<br>(patients without MAJOR TRAUMA)	6 studies<br>(N=10043)	cross-sectional (cohort type accuracy study)	Serious[a]	Not important	Serious[b]	Not important	None	555 (526 to 588)	⊕⊕◯◯<br>LOW
**False positives**<br>(patients incorrectly classified as having MAJOR TRAUMA)								175 (142 to 204)	

(a)Studies were downgraded by one increment for limitations in one risk of bias domain (patient selection)
(b)Studies were downgraded by one increment for inconsistency (was assessed by inspection of the sensitivity/specificity RevMan 5[2] plots)
(c)The judgement of precision for sensitivity and specificity separately was based on visual inspection of the confidence region in the diagnostic meta-analysis, where diagnostic metaanalysis has not been conducted imprecision was assessed using the confidence interval of the median sensitivity value. For studies with only AUC data precision was based on the corresponding 95%CI. Downgrading by one increment was applied for confidence intervals 10% or by two increments for confidence intervals more than 10%. If no variance data was available (imprecision could not be assessed) the studies were downgraded by one increment.

Tabella 2: Summary of findings. Diagnostic accuracy of MGAP in the prediction of major trauma in adults (standard of reference: in-hospital mortality)

Sensitivity (median)	0.90 (95% CI: 0.82 to 0.94)
Specificity (median)	0.79 (95% CI: 0.77 to 0.81)

Prevalences	6%

Outcome	№ of studies (№ of patients)	Study design	Factors that may decrease certainty of evidence					Effect per 1.000 patients tested	Test accuracy CoE
			Risk of bias	Indirectness	Inconsistency	Imprecision	Publication bias	pre-test probability of 6%	
**True positives**<br>(patients with MAJOR TRAUMA)	2 studi<br>262 pazienti	cross-sectional (cohort type accuracy study)	Not important	Not important	Not important	Serious[a]	None	54 (49 to 56)	⊕⊕⊕◯<br>MODERATE
**False negatives**<br>(patients incorrectly classified as not having MAJOR TRAUMA)								6 (4 to 11)	
**True negatives**<br>(patients without MAJOR TRAUMA)	2 studi<br>3999 pazienti	cross-sectional (cohort type accuracy study)	Not important	Not important	Not important	Not important	None	743 (724 to 761)	⊕⊕⊕⊕<br>HIGH
**False positives**<br>(patients incorrectly classified as having MAJOR TRAUMA)								197 (179 to 216)	

(a)The judgement of precision for sensitivity and specificity separately was based on visual inspection of the confidence region in the diagnostic meta-analysis, where diagnostic metaanalysis has not been conducted imprecision was assessed using the confidence interval of the median sensitivity value. For studies with only AUC data precision was based on the corresponding 95%CI. Downgrading by one increment was applied for confidence intervals 10% or by two increments for confidence intervals more than 10%. If no variance data was available (imprecision could not be assessed) the studies were downgraded by one increment.

Tabella 3: Summary of findings. Diagnostic accuracy of Triage - Revised Trauma Score T-RTS in the prediction of major trauma in adults (standard of reference: in-hospital mortality)

Sensitivity (median)	0.85 (95% CI: 0.77 to 0.91)	Prevalences	6%
Specificity (median)	0.61 (95% CI: 0.59 to 0.64)		

Outcome	№ of studies (№ of patients)	Study design	Factors that may decrease certainty of evidence					Effect per 1.000 patients tested	Test accuracy CoE
			Risk of bias	Indirectness	Inconsistency	Imprecision	Publication bias	pre-test probability of 6%	
**True positives** (patients with MAJOR TRAUMA)	2 studi (262 pazienti)	cross-sectional (cohort type accuracy study)	Not important	Not important	Not important	Serious[a]	None	51 (46 to 54)	⊕⊕⊕◯ MODERATE
**False negatives** (patients incorrectly classified as not having MAJOR TRAUMA)								9 (6 to 14)	
**True negatives** (patients without MAJOR TRAUMA)	2 studi (3999 pazienti)	cross-sectional (cohort type accuracy study)	Not important	Not important	Not important	Not important	None	578 (559 to 597)	⊕⊕⊕⊕ HIGH
**False positives** (patients incorrectly classified as having MAJOR TRAUMA)								362 (343 to 381)	

(a)The judgement of precision for sensitivity and specificity separately was based on visual inspection of the confidence region in the diagnostic meta-analysis, where diagnostic metaanalysis has not been conducted imprecision was assessed using the confidence interval of the median sensitivity value. For studies with only AUC data precision was based on the corresponding 95%CI. Downgrading by one increment was applied for confidence intervals 10% or by two increments for confidence intervals more than 10%. If no variance data was available (imprecision could not be assessed) the studies were downgraded by one increment.

Tabella 4: Summary of findings. Diagnostic accuracy of Pediatric Triage Tape tool in the prediction of major trauma in children (standard of reference: ISS >15)

Sensitivity (median)	0.36 (95% CI: 0.31 to 0.42)
Specificity (median)	0.75 (95% CI: 0.72 to 0.78)

Prevalences	22%

Outcome	№ of studies (№ of patients)	Study design	Factors that may decrease certainty of evidence					Effect per 1.000 patients tested	Test accuracy CoE
			Risk of bias	Indirectness	Inconsistency	Imprecision	Publication bias	pre-test probability of 22%	
**True positives** (patients with MAJOR TRAUMA)	2 studi 6936 pazienti	cross-sectional (cohort type accuracy study)	Serious[a]	Not important	Not important	Serious[c]	None	79 (68 to 94)	⨁⨁◯◯ LOW
**False negatives** (patients incorrectly classified as not having MAJOR TRAUMA)								141 (126 to 152)	
**True negatives** (patients without MAJOR TRAUMA)	2 studi 24639 pazienti	cross-sectional (cohort type accuracy study)	Serious[a]	Not important	Serious[b]	Not important	None	585 (562 to 608)	⨁⨁◯◯ LOW
**False positives** (patients incorrectly classified as having MAJOR TRAUMA)								195 (172 to 218)	

(a)Studies were downgraded by one increment for limitations in one risk of bias domain (index test)
(b)Studies were downgraded by one increment for inconsistency (was assessed by inspection of the sensitivity/specificity RevMan 5[2] plots)
(c)The judgement of precision for sensitivity and specificity separately was based on visual inspection of the confidence region in the diagnostic meta-analysis, where diagnostic metaanalysis has not been conducted imprecision was assessed using the confidence interval of the median sensitivity value. For studies with only AUC data precision was based on the corresponding 95%CI. Downgrading by one increment was applied for confidence intervals 10% or by two increments for confidence intervals more than 10%. If no variance data was available (imprecision could not be assessed) the studies were downgraded by one increment.

Tabella 5: Diagnostic accuracy of triage tools in the prediction of major trauma in adults

Index test	N studies	N patients	Risk of bias	Inconsitency[a]	Indirectness	Imprecision	Sensitivity % (median/ CI 95%)	Specificity % (median/ CI 95%)	Test accuracy CoE
**Reference standard: ISS >15**									
**TRENAU**	1	2572	Not important	None	None	None	0.92 (0.90 to 0.93)	0.41 (0.39 to 0.44)	HIGH
**Vittel Triage Criteria**	1	1160	Not important	None	None	None	NA	0.36 (0.32 to 0.40)	HIGH
**New Trauma team activation criteria**	1	998	Serious[b]	None	None	None	NA	0.13 (0.11 to 0.15)	MODERATE
**Reference standard: in-hospital mortality**									
**NTS (New Trauma Score)**	1	1001	Not important	None	None	Serious[c]	0.82 (0.71 to 0.90)	0.86 (0.84 to 0.88)	MODERATE
**Vittel triage Criteria**	1	1001	Not important	None	None	None	(0.95 to 1)	0.02 (0.01 to 0.03)	HIGH

(a) Not applicable (one study)

(b)Studies were downgraded by one increment for limitations in one risk of bias domain (patient selection)

(c)The judgement of precision for sensitivity and specificity separately was based on visual inspection of the confidence region in the diagnostic meta-analysis, where diagnostic metaanalysis has not been conducted imprecision was assessed using the confidence interval of the median sensitivity value. For studies with only AUC data precision was based on the corresponding 95%CI. Downgrading by one increment was applied for confidence intervals 10% or by two increments for confidence intervals more than 10%. If no variance data was available (imprecision could not be assessed) the studies were downgraded by one increment.

Tabella 6: Diagnostic accuracy of triage tools in the prediction of major trauma in children

Index test	N studies	N patients	Risk of bias	Inconsitency[a]	Indirectness	Imprecision	Sensitivity % (median/ CI 95%)	Specificity % (median/ CI 95%)	Test accuracy CoE
**Reference standard: ISS >15**									
**London**	1	701	Serious[b]	None	None	None	0.96 (0.92-0.98)	0.28 (0.24-0.33)	MODERATE
**East Midlands**	1	701	Serious[b]	None	None	None	0.97 (0.93-0.99)	0.17 (0.14- 0.21)	MODERATE
**North West**	1	701	Serious[b]	None	None	None	0.93 (0.89-0.96)	0.20 (0.17-0.24)	MODERATE
**Northern**	1	701	Serious[b]	None	None	None	0.91 (0.87-0.95)	0.23 (0.19-0.27)	MODERATE
**South West London**	1	701	Serious[b]	None	None	None	0.88 (0.83-0.92)	0.41 (0.37-0.46)	MODERATE
**Wessex**	1	701	Serious[b]	None	None	None	0.77 (0.71-0.83)	0.47 (0.43-0.52)	MODERATE
**Care Flight**	1	31292	Not important	None	None	None	0.64 (0.63 – 0.66)	0.90 (0.89-0.90)	HIGH
**JumpSTART/START**	1	31292	Not important	None	None	None	0.60 (0.58-0.61)	0.76 (0.76 – 0.77)	HIGH
**Triage Sort**	1	31292	Not important	None	None	None	0.71 (0.69-0.72)	0.78 (0.78-0.79)	HIGH
**Reference standard: in-hospital mortality/survival**									
**CareFlight**	1	31292	Not important	None	None	None	0.95 (0.94-0.97)	0.80 (0.80-0.81)	HIGH
**JumpSTART/START**	1	31292	Not important	None	None	None	0.92 (0.90-0.93)	0.70 (0.70-0.71)	HIGH
**Paediatric Triage Tape**	1	31292	Not important	None	None	None	0.38 (0.35-0.41)	0.66 (0.65-0.67)	HIGH
**Triage Sort**	1	31292	Not important	None	None	None	0.96 (0.95-0.97)	0.70 (0.69-0.70)	HIGH

(a) Not applicable (one study)
(b) Studies were downgraded by one increment for limitations in one risk of bias domain (patient selection, index test)

Supplement G. TREANU pre-hospital tool

The grading system uses criteria based on physiological findings, anatomical regions affected and mechanisms of injury, as described by the field triage decision scheme of the ACSCOT. Additionally, the TRENAU grading system incorporates the responses to treatment during the pre-hospital resuscitation. Each patient is graded as one of three levels of clinical severity, that is, A, B or C, adapted from the French Vittel triage criteria. This categorization permitted the allocation of each patient to the most suitable trauma center according to the TRENAU algorithm.

Vittel Criteria

Step 1 (Physiological signs)
GCS < 13
SAP < 90 mmHg
SpO2 < 90%

Step 2 (Global assessment of speed and mechanism)
Ejection from vehicle
Death in same passenger compartment
Fail > 6 m
Victim thrown or projected
Global assessment of speed and potential injuries :
Vehicle deformation, estimated vehicle spped no helmet, no seat belt
Blast

Step 3 (Anatomical injuries)
Penetrating trauma of head, neck, thorax, abdomen, arms or legs
Flail chest
Severe burn
Pelvic fracture
Suspicion of spinal cord injury
Amputation at or above wrist or ankle level
Acute limb ischemia

Step 4 (resuscitation)
Mechanical ventilation
Intravascular filling > 1000 ml
Vasopressor

Grade A: *instable despite resuscitation*

- Systolic arterial pressure < 90 mmHg

despite the use of vasopressors and more than 1L crystalloid fluids and/or a pre-hospital blood transfusion

- SpO2 < 90%

despite the use of mechanical ventilation or the use of facial mask with high-flow oxygen

Grade B: *stablilized after prehospital resuscitation or anatomic criteria*

- Systolic arterial pressure > 90 mmHg or SpO2 > 90% after initial resuscitation
- Isolated traumatic brain injury GCS <13 or glasgow motor response score < 5
- Suspicion of spinal cord injury
- Multiple thoracic fractures and flail chest
- Severe pelvic trauma
- Penetrating injury
- Amputation or crushed limb

Grade C: *Stable with high-kinetic circumstances or medical history*

- Fall from more than 6 meters
- Ejected/Projected/Blasted victim
- Death in same passenger compartment
- Assessment of speed accident: vehicle deformation, no seat belt, no helmet
- Medical history: <5 yrs or > 65 yrs, pregnancy, coagulation disorders

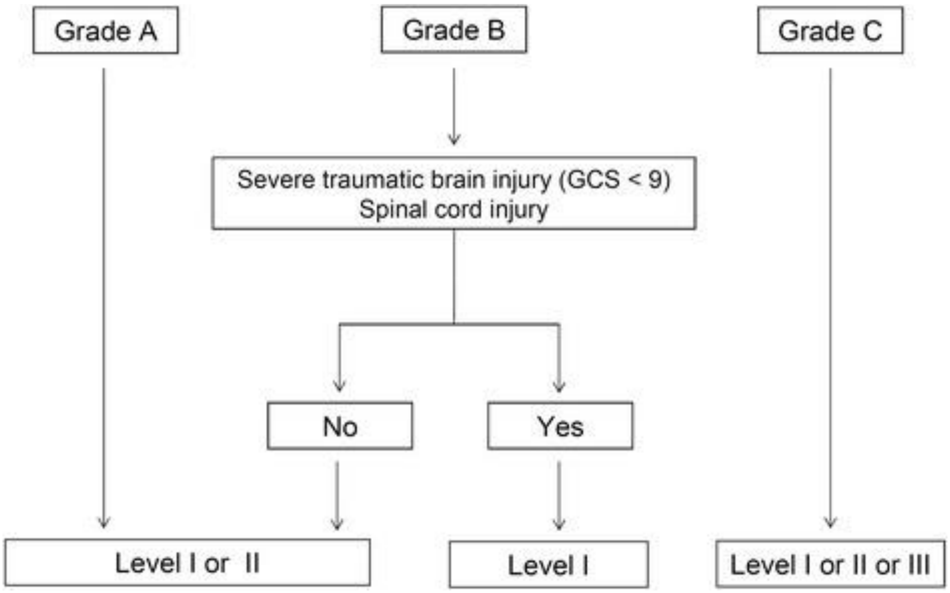

Categorization of trauma centres in the French North Alpine Trauma network (TRENAU).

Level	Available resources
Level I	24/7: Emergency room, intensive care unit, all specialized surgeries, interventional radiology, mass transfusion
Level II	24/7: Emergency room, intensive care unit, general surgery, conventional radiology with CT scan and interventional radiology, mass transfusion
Level IIII	24/7: Emergency room and conventional radiology with CT scan
